# Combined high leaf hydraulic safety and efficiency provides drought tolerance in *Caragana* species adapted to low mean annual precipitation

**DOI:** 10.1111/nph.16845

**Published:** 2020-09-07

**Authors:** Guang‐Qian Yao, Zheng‐Fei Nie, Neil C. Turner, Feng‐Min Li, Tian‐Peng Gao, Xiang‐Wen Fang, Christine Scoffoni

**Affiliations:** ^1^ State Key Laboratory of Grassland Agro‐ecosystems School of Life Sciences Lanzhou University Lanzhou 730000 China; ^2^ The UWA Institute of Agriculture and UWA School of Agriculture and Environment The University of Western Australia M082, 35 Stirling Highway Crawley WA 6009 Australia; ^3^ The Engineering Research Center of Mining Pollution Treatment and Ecological Restoration of Gansu Province Lanzhou City University Lanzhou 730070 China; ^4^ Department of Biological Sciences California State University Los Angeles 5151 State University Drive Los Angeles CA 90032 USA

**Keywords:** abscisic acid (ABA), *Caragana* species, drought, gas exchange, leaf hydraulics, turgor loss point, vein

## Abstract

- Clarifying the coordination of leaf hydraulic traits with gas exchange across closely‐related species adapted to varying rainfall can provide insights into plant habitat distribution and drought adaptation.
- The leaf hydraulic conductance (*K*_leaf_), stomatal conductance (*g*_s_), net assimilation (*A*), vein embolism and abscisic acid (ABA) concentration during dehydration were quantified, as well as pressure–volume curve traits and vein anatomy in 10 *Caragana* species adapted to a range of mean annual precipitation (MAP) conditions and growing in a common garden.
- We found a positive correlation between Ψ_leaf_ at 50% loss of *K*_leaf_ (*K*_leaf_ *P*_50_) and maximum *K*_leaf_ (*K*_leaf‐max_) across species. Species from low‐MAP environments exhibited more negative *K*_leaf_ *P*_50_ and turgor loss point, and higher *K*_leaf‐max_ and leaf‐specific capacity at full turgor, along with higher vein density and midrib xylem per leaf area, and a higher ratio of *K*_leaf‐max_ : maximum *g*_s_. Tighter stomatal control mediated by higher ABA accumulation during dehydration in these species resulted in an increase in hydraulic safety and intrinsic water use efficiency (WUE_i_) during drought.
- Our results suggest that high hydraulic safety and efficiency combined with greater stomatal sensitivity triggered by ABA production and leading to greater WUE_i_ provides drought tolerance in *Caragana* species adapted to low‐MAP environments.

## Introduction

Leaf hydraulic conductance (*K*_leaf_) decreases with soil water potential during drought stress, and/or in response to high evaporative demand due to high vapour pressure deficit (Scoffoni *et al*., 2012; Baer *et al*., 2016; Brodribb *et al*., 2016; Sack *et al*., 2016; Trifiló *et al*., 2016; Scoffoni & Sack, 2017). As a result, it has been suggested that decreasing *K*_leaf_ induces stomatal closure and thus decreases photosynthetic rates, impacting plant growth (Blackman *et al*., 2009; Scoffoni *et al*., 2018; Wang *et al*., 2018; Trueba *et al*., 2019). Thus, maintaining leaf hydraulic function during soil and/or atmospheric drought can be an important feature which enables a species to thrive, particularly in low mean annual precipitation (MAP) environments (Blackman *et al*., 2012, 2014; Brodribb *et al*., 2014; Nardini & Luglio, 2014).

The leaf water potential (Ψ_leaf_) at 50% loss of *K*_leaf_ (*K*_leaf_ *P*_50_) has been widely used as an indicator of species resistance to leaf hydraulic decline (Scoffoni & Sack, 2017). However, only three studies, to our knowledge, have investigated the link between *K*_leaf_ *P*_50_ and climate. Across diverse species in Australian forests, *K*_leaf_ *P*_50_ was more negative in habitats with low precipitation (or high aridity) (Blackman *et al*., 2012, 2014). Additionally, a weak though significant positive correlation between *K*_leaf_ *P*_50_ and MAP was found across a meta‐analysis of 150 diverse species from sclerophyllous, temperate and tropical forests (Nardini & Luglio, 2014). Similarly, such trends have been observed at the stem level, with the water potential at 50% loss of stem hydraulic conductance (*K*_stem_ *P*_50_) strongly predicting species’ distribution across water availability gradients at local and global scales (Maherali *et al*., 2004; Choat *et al*., 2007; Choat *et al*., 2012). However, no studies to our knowledge have investigated how variation in *K*_leaf_ vulnerability relates to habitat MAP across closely related species.

Past studies have shown a tradeoff between leaf hydraulic safety (*K*_leaf_ *P*_50_) and efficiency (maximum leaf hydraulic efficiency, *K*_leaf‐max_; Scoffoni *et al*., 2012; Nardini & Luglio, 2014; Scoffoni & Sack, 2017). Thus, if species from low‐MAP environments in their native habitats have more negative *K*_leaf_ *P*_50_, this tradeoff implies they would also have lower *K*_leaf‐max_. However, some studies have found that species growing in drier and/or warmer climates have a higher *K*_leaf‐max_ compared to maximum stomatal conductance (*g*_s‐max_) to replace water loss and mitigate the greater evaporative loss (Brodribb & Jordan, 2008; Scoffoni *et al*., 2016, 2015). Therefore, it might be more adaptive for species to display both high hydraulic safety and high hydraulic efficiency in low‐MAP environments. But no study to our knowledge has investigated the coordination across species growing along a precipitation gradient. We hypothesized that species from low‐MAP environments would have more negative *K*_leaf_ *P*_50_ to tolerate low‐MAP climates, and higher hydraulic supply and, thus, higher ratios of *K*_leaf‐max_ : *g*_s‐max_.

To better understand the association or coordination between traits, it is essential to elucidate the drivers behind physiological level trait diversity. Therefore, we also investigated the anatomical and biochemical determinants behind the trait differences observed across species from low‐ to high‐MAP environments. If species from low‐MAP habitats have more negative *K*_leaf_ *P*_50_ and higher *K*_leaf‐max_ as hypothesized, how are they achieving these values? We first tested for a positive correlation between *K*_leaf‐max_ and major and minor vein length per unit area (VLA), midrib xylem area per leaf area, and leaf‐specific capacitance at full turgor (C*_FT_). Indeed, studies have shown that an increase in VLA can cause an increase in leaf xylem hydraulic conductance and outside‐xylem hydraulic conductance (Scoffoni *et al*., 2011; Buckley *et al*., 2015; Scoffoni & Sack, 2017), that greater midrib xylem conduit sizes are related to high *K*_leaf‐max_ (Nardini *et al*., 2012), and that an increase in C*_FT_ contributes to high *K*_leaf‐max_ (Sack *et al*., 2003; Xiong & Nadal, 2020). Using the optical method to record spatial and temporal patterns of embolism formation in the veins of dehydrated leaves (Brodribb *et al*., 2016), we tested whether the decrease observed in *K*_leaf_ with dehydration was caused by vein xylem embolism (*K*_x_), or by changes in outside‐xylem pathways (*K*_ox_) (Brodribb *et al*., 2016; Scoffoni *et al*., 2017), and/or whether it was linked to turgor loss point (π_tlp_) (Scoffoni *et al*., 2014), which has also been shown to be a strong indicator of species tolerance to low‐MAP or arid environments (Bartlett *et al*., 2012).

To understand the response of stomata to dehydration, we investigated the role of foliar abscisic acid (ABA) accumulated during dehydration on stomatal closure (Brodribb & McAdam, 2013), and whether the difference in rates of ABA synthesis during dehydration across species could lead to differential hysteresis in stomatal closure (Seo & Koshiba, 2002; Lovisolo *et al*., 2008; Brodribb & McAdam, 2013; McAdam & Brodribb, 2015). Furthermore, if ABA synthesis causes early stomatal closure during dehydration, this could lead to increasing leaf intrinsic water use efficiency (net carbon assimilation/stomatal conductance, WUE_i_) (Negin & Moshelion, 2016).

Comparing hydraulic characteristics across distantly related species versus closely‐related species can give different, even strongly opposing, conclusions; therefore, to establish a true association of responses, tests must be made using closely‐related plants in well‐resolved lineages (Gleason *et al*., 2016; Scoffoni *et al*., 2016). In addition, common garden experiments, which maximally reduce trait plasticity, are useful in exposing genetic variation (Scoffoni *et al*., 2016; Jankowski *et al*., 2019; Ignazia *et al*., 2020). Therefore, in order to establish associations between leaf hydraulic traits and gas exchange, we investigated the hydraulic safety–efficiency trade‐off, the correlation between *K*_leaf‐max_ and *g*_s‐max_ (*K*_leaf_), and WUE_i_ during dehydration in 10 *Caragana* species adapted to a range of MAP, from < 200 mm to > 1400 mm and growing in a common garden. We hypothesized that species from low‐MAP environments would display hydraulic strategies prioritizing hydraulic safety and would therefore exhibit the following characteristics: more negative *K*_leaf_ *P*_50_ and higher *K*_leaf‐max_, yielding a positive correlation between hydraulic safety and hydraulic efficiency across species; greater leaf WUE_i_ driven by tighter stomatal control during dehydration as a result of a greater accumulation of ABA; more negative *K*_leaf_ *P*_50_ associated with a more negative π_tlp_ rather than resistance to embolism formation in the leaf xylem; higher vein density, midrib xylem area per leaf area and leaf‐specific capacitance driving greater *K*_leaf‐max_ values.

## Materials and Methods

### Plant materials

*Caragana* is a deciduous genus of great ecological and economical value in East Asia. Indeed, *Caragana* species dominate both the arid shrublands and temperate forests of East Asia (Zhang *et al*., 2009, 2015; Fang *et al*., 2017). Furthermore, species from this genus are widely used in the restoration of degraded land by fixing atmospheric nitrogen, forming shelterbelts for crops and pastures, providing supplemental livestock forage (leaves and flowers) and fuel energy (shoots) for local farmers, and assisting in the conservation of soil and water (Zhang *et al*., 2009, 2015; Fang *et al*., 2017). They are often selected for vegetation rehabilitation in returning farmland to woodland, and contribute to increasing forest cover in China (Fang *et al*., 2017; Chen *et al*., 2019). In this study, we collected 10 *Caragana* species (*Caragana korshinskii*, *Caragana* *tibetica*, *Caragana* *roborovskyi*, *Caragana intermedia*, *Caragana* *microphylla*, *Caragana* *opulens*, *Caragana* *arborescens*, *Caragana boisi*, *Caragana* *stipitata*, *Caragana* *sinica*) growing in regions varying between 110 and 1400 mm in MAP, and from 0.68 to 32.2 in aridity index (AI = potential evapotranspiration/MAP; Supporting Information Table S1). Notably, mean annual temperature (MAT) displays little variation throughout these regions, ranging from 2.6°C to 9.0°C (data from the China Meteorological Data Sharing Service System, http://data.cma.cn/; Table S1). While precipitation as snow in winter contributes to the soil water supply in spring as the snow melts, > 70% of the total annual precipitation occurs in the areas of collection as rainfall during the growing season from May to October; hereafter for convenience, we refer to species as growing in ‘low‐MAP’ (≤ 400 mm MAP) and ‘high‐MAP’ (≥ 400 mm) environments.

Seeds of the first nine species were collected as described by Fang *et al*. (2017). *Caragana* *sinica* is triploid and has no seeds, and tissue was cultured from > 100 individuals as described by Song *et al*. (2007). For details see Methods S1. The plants were grown in a common garden to disentangle genetic differences from environmentally‐based ones in a naturally‐lit glasshouse on the Yuzhong campus (lat. 35°51′N, long. 104°07′E; altitude 1620 m) of Lanzhou University, Lanzhou, Gansu Province, China.

### Drought stress

On 1 June 2014, 16‐month‐old plants (*n* = 40) of each species with a height of 0.5–1.0 m and with > 120 compound leaves or palmate leaves were selected. Leaflets were taken to determine leaf dry mass per unit area (LMA) and anatomical traits, and shoots were removed to determine parameters of pressure–volume curves (see subsection ‘Leaf water relations from pressure–volume curves’, below). Then drought stress treatments were imposed: water was withheld such that every day over the first 10 d, individuals were re‐watered with one‐half of the volume of water transpired during the preceding day, by weight (Brodribb & McAdam, 2013). After 10 d, water was withheld completely for the next 40 d at which point the experiment was terminated. Every 1–3 d as the pots dehydrated, the predawn leaf water potential (Ψ_leaf_) and the leaf hydraulic conductance (*K*_leaf_) were measured on the 10 study species to construct vulnerability curves. For a subset of six species (*C. korshinskii*, *C. intermedia*, *C. microphylla*, *C. boisi*, *C. stipitata* and *C. sinica*) selected to span the precipitation gradient, the gas exchange rates were measured every 1–3 d as the pots dehydrated. After each gas exchange measurement, the leaf was cut off the plant and the leaflets which were not used during the gas exchange measurement were divided in two. Half were weighed immediately and then dried to constant mass to determine the dry mass/fresh mass ratio, and the other half was also weighed immediately then placed in liquid nitrogen and stored in a freezer at −80°C to measure ABA content.

### *K*_leaf_ measurement

*K*_leaf_ was measured between 04:30 and 06:30 h Beijing Standard Time (BST) using the evaporative flux method (EFM) after each predawn Ψ_leaf_ measurement (between 04:30 and 05:30 h BST Sack *et al*., 2002; Scoffoni *et al*., 2011; Sack & Scoffoni, 2012). Instead of constructing *K*_leaf_ vulnerability curves from detached dehydrated branches, as is typically done, we constructed these from the dehydrating plants. Thus, throughout the 50‐day dehydration period, two leaves were excised at predawn to measure the initial Ψ_leaf_ using a pressure chamber (Plant Moisture Stress model 1000; PMS Instrument Co., Albany, OR, USA). A third leaf (typically the middle leaf) was excised immediately under double distilled water, and was connected to silicone tubing to determine the transpiration rate under a light source (1000 μmol m^2^ s^−1^ photosynthetically active radiation). The final Ψ_leaf_ (the driving force of the flow rate) was determined using the pressure chamber (Sack *et al*., 2002; Scoffoni *et al*., 2011; Sack & Scoffoni, 2012) at the end of the *K*_leaf_ measurement. The vulnerability curve (the change in *K*_leaf_ with Ψ_leaf_) was obtained by plotting *K*_leaf_ against the most negative Ψ_leaf_ (in our data this was always the predawn Ψ_leaf_) (Sack & Scoffoni, 2012).

### Gas exchange measurements

Stomatal conductance (*g*_s_) and the net assimilation rate (*A*) were measured on one upper fully‐expanded leaf adjacent to the leaf used for *K*_leaf_ in the morning (07:00 and 08:30 h BST) using a portable open gas‐exchange system (LI‐6400, LiCor, Lincoln, NE, USA). The gas exchange chamber was supplied with a photosynthetic photon flux density of 1200 μmol m^−2^ s^−1^ provided by an LED source. Leaf temperature was maintained at 22 °C, CO_2_ was set at 400 ppm, and vapor‐pressure deficit (VPD) was set at about 1.5 kPa. The time of measurement was chosen as early morning because preliminary data showed that gas exchange values for the *Caragana* species were highest during those hours. Both *g*_s_ and *A* were measured as the pots dehydrated (on four leaves from different individuals per species per d of measurement) for the six subsampled species listed in the ‘Drought stress’ subsection, above. Additionally, for the remaining four species, *g*_s‐max_ and *A*_max_ were measured in the well‐watered control pots (on four leaves from different individuals per species per d for 3 d in succession).

### ABA determination

Leaf ABA concentration was determined following a previously described method (McAdam, 2015). The frozen leaf material was ground in a mortar with liquid nitrogen, and the ground sample was extracted with 5 ml of methanol/water (1 : 1, v/v, pH = 3 with formic acid) and left overnight at 4°C with added labelled ABA (D6‐ABA). The next day, samples were centrifuged and the supernatant was removed. The remaining pellets were extracted with methanol/water twice and the supernatants were combined. The collected supernatant was extracted with petroleum ether, extracted with an ester phase and dried under nitrogen gas using a Termovap Sample Concentrator (HP5106GD; Shanghai Eastern Analytical Instrument Co. Ltd, Shanghai, China). The residue was dissolved in chromatographic purity methanol for analysis with an ultra‐high performance liquid chromatography tandem mass spectrometer (OrbiTrap Fusion Lumos; Thermo Fisher, San Jose, CA, USA). The ABA concentration (ng g^−1^ dry weight) was calculated as the ABA concentration per leaf fresh mass × (leaf dry mass/leaf fresh mass) of adjacent leaflets to the leaflet sampled for ABA.

### Leaf water relations from pressure–volume curves

Six shoots from different individuals of eight species (excluding *C. tibetica and C. opulens* with short petioles) were removed in the evening before the drought experiment and rehydrated overnight (Ψ_leaf_ > −0.3 MPa) to construct pressure–volume curves. One leaf of each shoot per species was removed and immediately scanned to calculate leaf area, and then Ψ_leaf_ and leaf mass were repeatedly measured as they dehydrated on the bench. Following standard pressure–volume curve analysis, the osmotic pressure at full turgor (π_o_), osmotic pressure at turgor loss point (π_tlp_), modulus of elasticity (ɛ), relative water content at turgor loss point (RWC_TLP_), saturated water content per unit dry mass (SWC), and the area‐based leaf‐specific capacity at full turgor (C*_FT_) were calculated (Turner, 1988; Sack *et al*., 2011).

### Optical method

The optical method, which records spatial and temporal patterns of embolism formation in the veins of water‐stressed leaves (Brodribb *et al*., 2016), was used to determine vein embolism in the subset of six *Caragana* species. Cumulative embolism in 1° and 2° veins (embolized area/the total cumulative embolized area × 100%) was analyzed (see http://www. opensourceov.org for the detailed image analysis protocol) using a digital camera (EOS 5D Mark III; Canon, Tokyo, Japan) attached to a microscope (Ex30LED; Sunny Instruments, Ningbo, China). The majority of 3° veins and all high‐order veins were difficult to observe as these typically do not have bundle sheath extensions allowing light penetration. For details see Methods S1. The Ψ_leaf_ at which leaf conduits reached 12% cumulative embolism (quantifying the initial embolism formation; *PLC*_major_ *P*_12_) and 50% cumulative embolism (an index of resistance to embolism formation; *PLC*_major_ *P*_50_) in 1° and 2° vein orders (quantified by changes in pixel coloration as described in Methods S1) were obtained for three leaflets from different individuals per species.

### Leaf mass per area and anatomical traits

Leaflets removed from well‐watered plants were scanned at 300 dpi and then oven‐dried at 70°C for 48 h, after which their dry mass was determined and LMA was calculated. Both major and minor vein lengths per area were determined following the method as described by Sack *et al*. (2002) after leaflets were cleared in 5% sodium hydroxide. Midrib xylem anatomical traits were determined on leaf cross‐sections obtained using a freeze‐microtome as described by Blackman *et al*. (2010) and Fang *et al*. (2014). We measured the entire area of the midrib xylem in cross sections using Imagej software (https://imagej.nih.gov/ij/). Midrib xylem area was then standardized per leaflet area. For details see Methods S1.

### Statistical analyses

Differences between means of parameters across species were evaluated by one‐way ANOVAs (Duncan’s multiple range test), and differences between species from low‐ vs high‐MAP environments were evaluated with unpaired *t*‐tests. All statistical analyses were performed with Spss 15.0 (SPSS Inc., Chicago, IL, USA), and results were considered significant at *P* < 0.05. To construct hydraulic vulnerability curves, we selected the maximum likelihood function that best fitted our data for each species using the *optim* function in R 3.1.0 (https://www.r‐project.org/). Four functions were tested as described by Scoffoni *et al*. (2011): a linear function (*K*z = aΨ_z_+b), a three‐parameter sigmoidal function $\left(Kz=\frac{a}{1+e^{‐\left(\frac{\psi_{z}‐x_{0}}{b}\right)}}\right)$, a logistic function $\left(Kz=\frac{a}{1+{\left(\frac{\psi_{z}}{x_{0}}\right)}^{b}}\right)$, and an exponential function $\left(Kz=y_{0}+ae^{‐b\psi_{z}}\right)$. The *K*z in the above functions represents either the *K*_leaf_, *g*_s_, or net assimilation (*A*), and Ψ_z_ represents leaf water potential. Functions were compared using the Akaike Information Criterion (AIC) corrected for low *n*. The function with the lowest AIC value (differences of > 2 considered) was chosen as the maximum likelihood function. The maximum and *P*_50_ values were calculated from the best fit function for all three traits. For the four species for which *g*_s_ and *A* vulnerability curves were not obtained, averages from leaves of fully hydrated plants were calculated to obtain maximum *g*_s_ (*g*_s‐max_) and maximum *A* (*A*_max_). Trait–trait correlations were performed using the linear, polynomial (inverse first order), peak (gaussian) or sigmoidal function of sigmaplot 10.0 (Systat Software Inc., San Jose, CA, USA).

### Data availability

The data for the morphological, anatomical and physiological traits that support the findings of this study are available in the form of a supplementary Excel file. Additional information is available from the corresponding authors upon reasonable request.

## Results

### Leaf hydraulic efficiency and safety

**Fig. 1:**
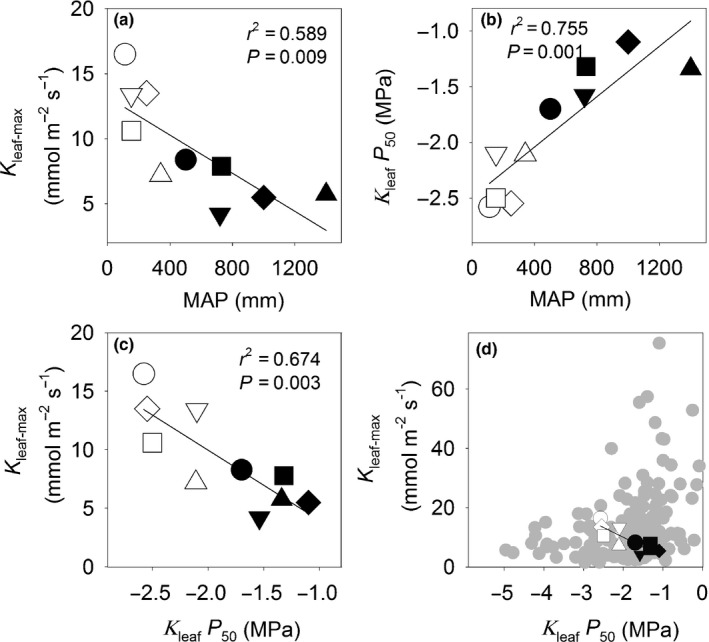
Hydraulic efficiency and safety. The relationships between (a) maximum leaf hydraulic conductance (*K*_leaf‐max_) and mean annual precipitation (MAP), (b) the leaf water potential at 50% loss of leaf hydraulic conductance (*K*_leaf_ *P*_50_) and MAP, and (c) *K*_leaf‐max_ and *K*_leaf_ *P*_50_ across 10 *Caragana* species. The data from (c) falls within (d) the relationship between *K*_leaf‐max_ and *K*_leaf_ *P*_50_ obtained for 137 angiosperm species in a recent meta‐analysis (grey symbols) (Scoffoni & Sack, 2017). The correlation coefficient (*r* ^2^) and probability (*P*) of the fitted linear regressions are given. Open symbols represent species from low‐MAP environments, and closed symbols represent species from high‐MAP environments: *C. arborescens*, closed inverted triangles; *C. boisi*, closed squares; *C. intermedia*, open diamonds; *C. korshinskii*, open circles; *C. microphylla*, open triangles; *C. opulens*, closed circles; *C. roborovskyi*, open squares; *C. sinica*, closed triangles; *C. stipitata*, closed diamonds; *C. tibetica*, inverted open triangles.

The *Caragana* species growing in a common garden showed fourfold variation in *K*_leaf‐max_ (a measure of hydraulic efficiency), from 16.5 mmol m^−2^ s^−1^ in *C. korshinskii,* a species from low‐MAP environments (< 400 mm), to 4.2 mmol m^−2^ s^−1^ in *C. arborescens,* a species from high‐MAP environments (> 400 mm). Species from low‐MAP environments had significantly higher *K*_leaf‐max_ than species from high‐MAP environments (*t*‐test, *P* = 0.011). *K*_leaf‐max_ was negatively correlated with MAP across species (Fig. 1a). With decreasing predawn Ψ_leaf_, *K*_leaf_ decreased gradually, but in the five species from low‐MAP environments, *K*_leaf_ was less sensitive to decreasing predawn Ψ_leaf_ than in the five species from high‐MAP environments (Fig. S1), so that *K*_leaf_ *P*_50_ (a measure of hydraulic safety) was lower (more negative) in the species from low‐MAP environments than those from high‐MAP environments (*t*‐test, *P* < 0.001) and was positively correlated to original habitat MAP across species (Fig. 1b). We found a significant positive relationship between hydraulic safety (more negative *K*_leaf_ *P*_50_) and hydraulic efficiency (higher *K*_leaf‐max_) (Fig. 1c). Notably, the range of *K*_leaf_ *P*_50_ and *K*_leaf‐max_ of *Caragana* species overlapped with the range observed across 137 species in a recent meta‐analysis (Scoffoni & Sack, 2017) (Fig. 1d), though it exhibited a contrasting trend.

### Gas exchange and water use efficiency

**Fig. 2:**
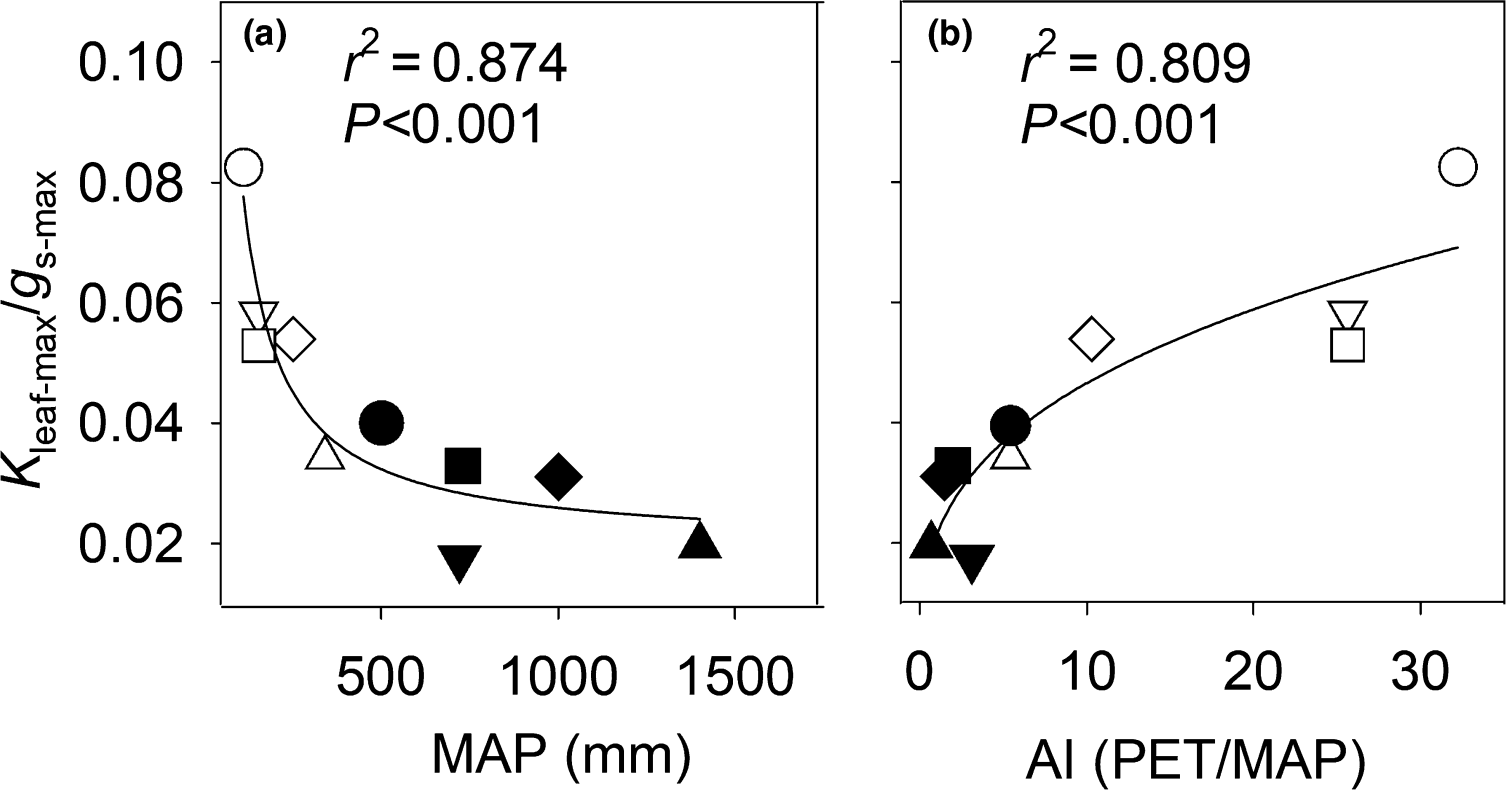
Correlation between hydraulic supply and demand with climate. The relationship between the ratio of maximum leaf hydraulic conductance (*K*_leaf‐max_) to maximum stomatal conductance (*g*_s‐max_) and mean annual precipitation (MAP) (a), and the aridity index (AI, potential evapotranspiration (PET)/MAP) (b), across 10 *Caragana* species. The correlation coefficients (*r* ^2^) and probabilities (*P*) for the fitted inverse first order regression (a) and logistic regression (b) are given. Open symbols represent species from low‐MAP environments, and closed symbols represent species from high‐MAP environments: *C. arborescens*, closed inverted triangles; *C. boisi*, closed squares; *C. intermedia*, open diamonds; *C. korshinskii*, open circles; *C. microphylla*, open triangles; *C. opulens*, closed circles; *C. roborovskyi*, open squares; *C. sinica*, closed triangles; *C. stipitata*, closed diamonds; *C. tibetica*, inverted open triangles.

In contrast to *K*_leaf‐max,_ *g*_s‐max_ showed only a 1.6‐fold variation across 10 *Caragana* species, from 0.18 mol m^−2^ s^−1^ in *C. stipitata* to 0.29 mol m^−2^ s^−1^ in *C. sinica* (Fig. S2; Table S2), and *g*_s‐max_ was not correlated with MAP (Fig. S3a) or *K*_leaf‐max_ (Fig. S3b). As a result, *K*_leaf‐max_/*g*_s‐max_ was significantly lower with increasing MAP and decreasing AI across species (Fig. 2a,b). After water was withheld, *g*_s_ of species from low‐MAP environments was more sensitive to the decrease in predawn Ψ_leaf_ than species from high‐MAP environments (Fig. S2). As a result, species from low‐MAP environments had less negative values of *g*_s_ *P*_50_ than species from high‐MAP environments (*t*‐test, *P* = 0.009; Fig. S4), and *g*_s‐max_ was not correlated with *g*_s_ *P*_50_ (*r* ^2^ = 0.011, *P* = 0.843). Similarly to *g*_s‐max_, *A*_max_ showed little variation (1.3‐fold), from 12.6 mmol m^−2^ s^−1^ in *C. stipitata* to 16.1 mmol m^−2^ s^−1^ in *C. intermedia*. No significant differences were found in *A*_max_ between species from low‐ vs high‐MAP environments (*t*‐test; *P* = 0.512), and *A*_max_ was not correlated with *K*_leaf‐max_ (*r* ^2^ = 0.025, *P* = 0.663) nor *g*_s‐max_ (*r* ^2^ = 0.205, *P* = 0.189). *A* decreased with decreasing predawn Ψ_leaf_ after water was withheld across six *Caragana* species, and *A*_max_ was not correlated with *A P*_50_ (*r* ^2^ = 0.07, *P* = 0.616).

**Fig. 3:**
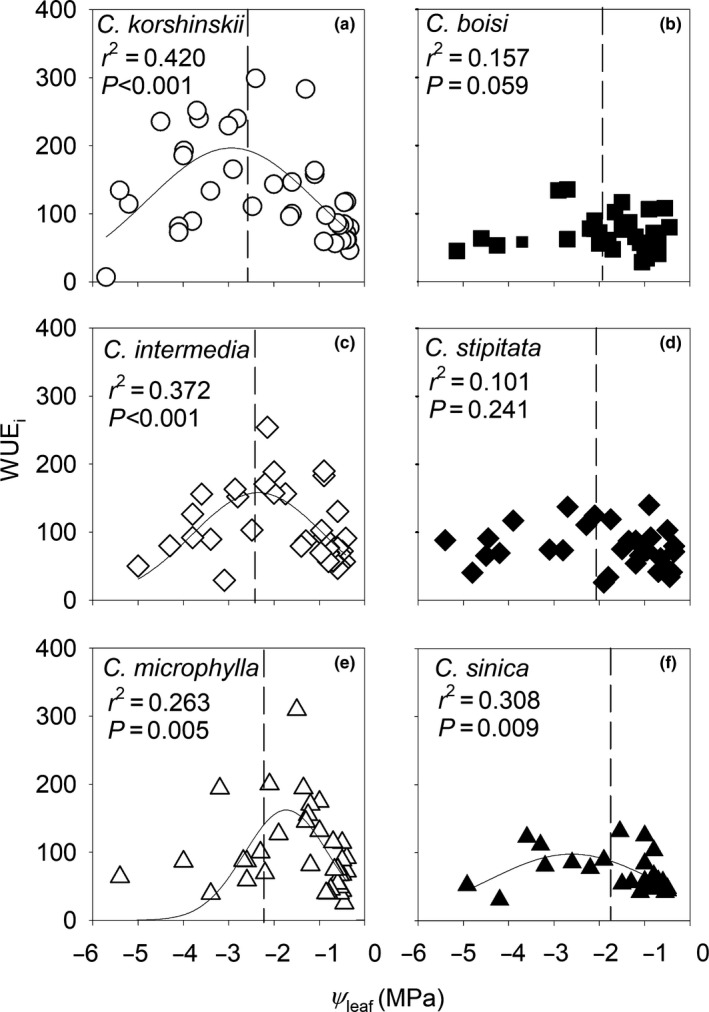
Drought‐induced intrinsic water use efficiency (WUE_i_, net photosynthesis/stomatal conductance). Relationship between WUE_i_ and predawn leaf water potential (Ψ_leaf_) in species from low mean annual precipitation (MAP) environments (a, c, e) and high‐MAP environments (b, d, f). The line indicates significant regressions (*P < *0.05), and the correlation coefficient (*r* ^2^) and probability (*P*) of the fitted Gaussian regressions are listed in (a, c, e, f). The vertical dashed line represents the turgor loss point (π_tlp_) from the pressure–volume curve for each species.

Notably, *A* was less sensitive to decreasing predawn Ψ_leaf_ than *g_s_* (Fig. S5), with *A P*_50_ values occurring −0.3 to −0.8 MPa below *g*_s_ *P*_50_ across species. This resulted in an increase in intrinsic leaf water use efficiency (WUE_i_ = *A*/*g*_s_) before π_tlp_ in species from low‐MAP environments, which showed greater sensitivity in *g*_s_ but not *A* (Fig. 3). Maximum WUE_i_ (calculated at the water potential when the value peaked across species; Fig. 3) was higher in species from low‐MAP environments (on average 200.2 ± 6.50) compared to species from high‐MAP environments (average 99.1 ± 2.72) (*t*‐test, *P* < 0.001). The WUE_i_ in species from low‐MAP environments was 2.6, 1.6, and 2.2‐fold higher at *K*_leaf_ *P*_50_, *g*_s_ *P*_50_ and π_tlp_, respectively, compared to species from high‐MAP environments. Notably, WUE_i_ values in well‐watered plants were similar across species, varying only 1.6‐fold from 44.8 to 73.7 (Table S2), and no correlation between *K*_leaf_ *P*_50_ and WUE_i_ of well‐watered plants were found (*r* ^2^ = 0.551, *P* = 0.091). However, significant correlations were found between *K*_leaf_ *P*_50_ and WUE_i_ at *K*_leaf_ *P*_50_, *g*_s_ *P*_50_ and π_tlp_ (*r* ^2^ = 0.816–0.960, *P* = 0.001–0.014).

### Drought tolerance traits and anatomical and biochemical drivers

**Fig. 4:**
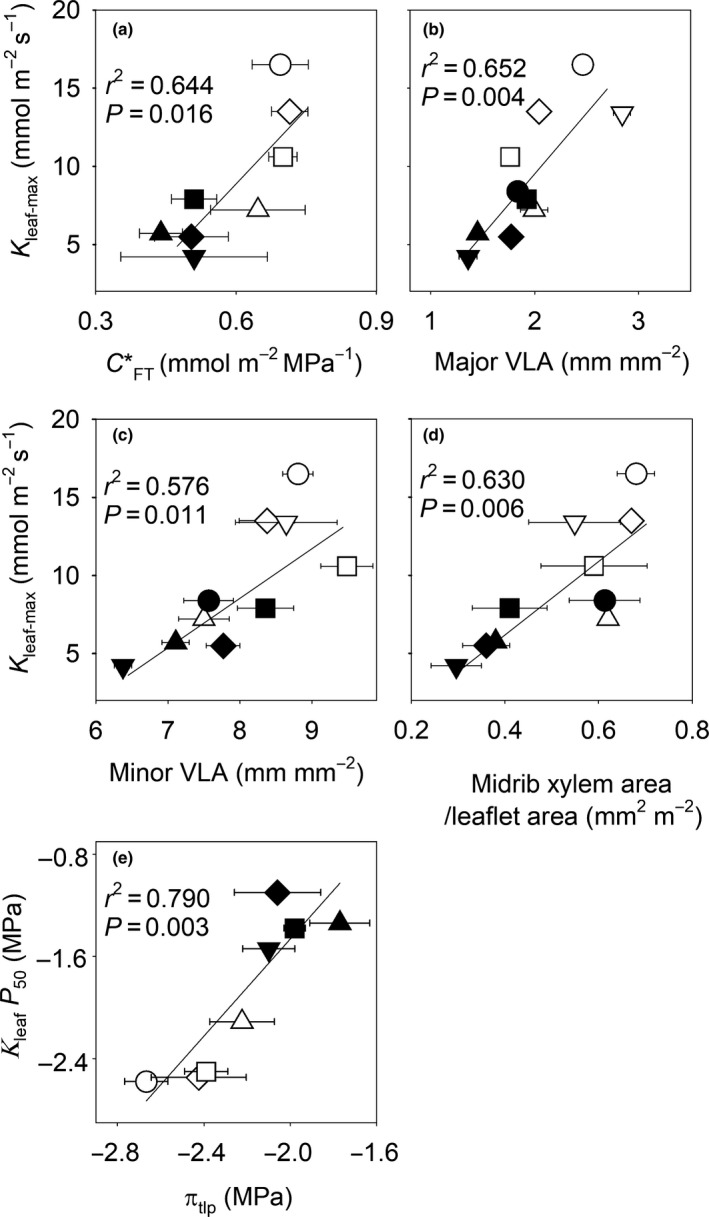
Relationship between maximum leaf hydraulic conductance, leaf‐specific capacity and leaf venation structure, and between the leaf water potential at 50% loss of leaf hydraulic conductance and turgor loss point. The relationship (a) between the maximum leaf hydraulic conductance (*K*_leaf‐max_) and area‐based leaf‐specific capacity at full turgor (C*_FT_) (*n* = 8), (b–d) between *K*_leaf‐max_ and major vein length per area (VLA) (b), minor VLA (c) and midrib xylem conduit area per leaflet area (d) (*n* = 10), and (e) between the leaf water potential at 50% loss of leaf hydraulic conductance (*K*_leaf_ *P*_50_) and osmotic potential at the turgor loss point (π_tlp_) (*n* = 8) in *Caragana* species. The correlation coefficients (*r* ^2^) and probabilities (*P*) of the fitted linear regressions are given. The bars are ± 1 SE of the mean (*n* = 6) when larger than the symbol. Open symbols represent species from low mean annual precipitation (MAP) environments, and closed symbols represent species from high‐MAP environments: *C. arborescens*, closed inverted triangles; *C. boisi*, closed squares; *C. intermedia*, open diamonds; *C. korshinskii*, open circles; *C. microphylla*, open triangles; *C. opulens*, closed circles; *C. roborovskyi*, open squares; *C. sinica*, closed triangles; *C. stipitata*, closed diamonds; *C. tibetica*, inverted open triangles.

Species varied by 1.2‐ to 1.6‐fold in their π_o_, π_tlp,_ SWC and C*_FT_ values (one‐way ANOVA; *P* = 0.001–0.045). Additionally, species from low‐MAP environments displayed more negative π_o_ and π_tlp_, and higher SWC and C*_FT_ than species from high‐MAP environments (*t*‐test; *P* = 0.005–0.008; Tables S2, S3). The modulus of elasticity (ɛ) and LMA did not differ across species (*P* = 0.436–0.706; Tables S2, S3). Significant variation was found in venation and cross‐sectional anatomical traits across species (one‐way ANOVA; *P* = 0.005–0.008). Notably, major, minor and total VLA, and midrib xylem area per leaflet area were higher in species from low‐MAP environments than in species from high‐MAP environments (*t*‐test; *P* = 0.008–0.04; Fig. S6). Significant correlations between *K*_leaf‐max_ and the C*_FT_, major VLA, minor VLA, and midrib xylem area per leaflet area were found (Fig. 4a–d), as well as between π_tlp_ and *K*_leaf_ *P*_50_ (Fig. 4e).

**Fig. 5:**
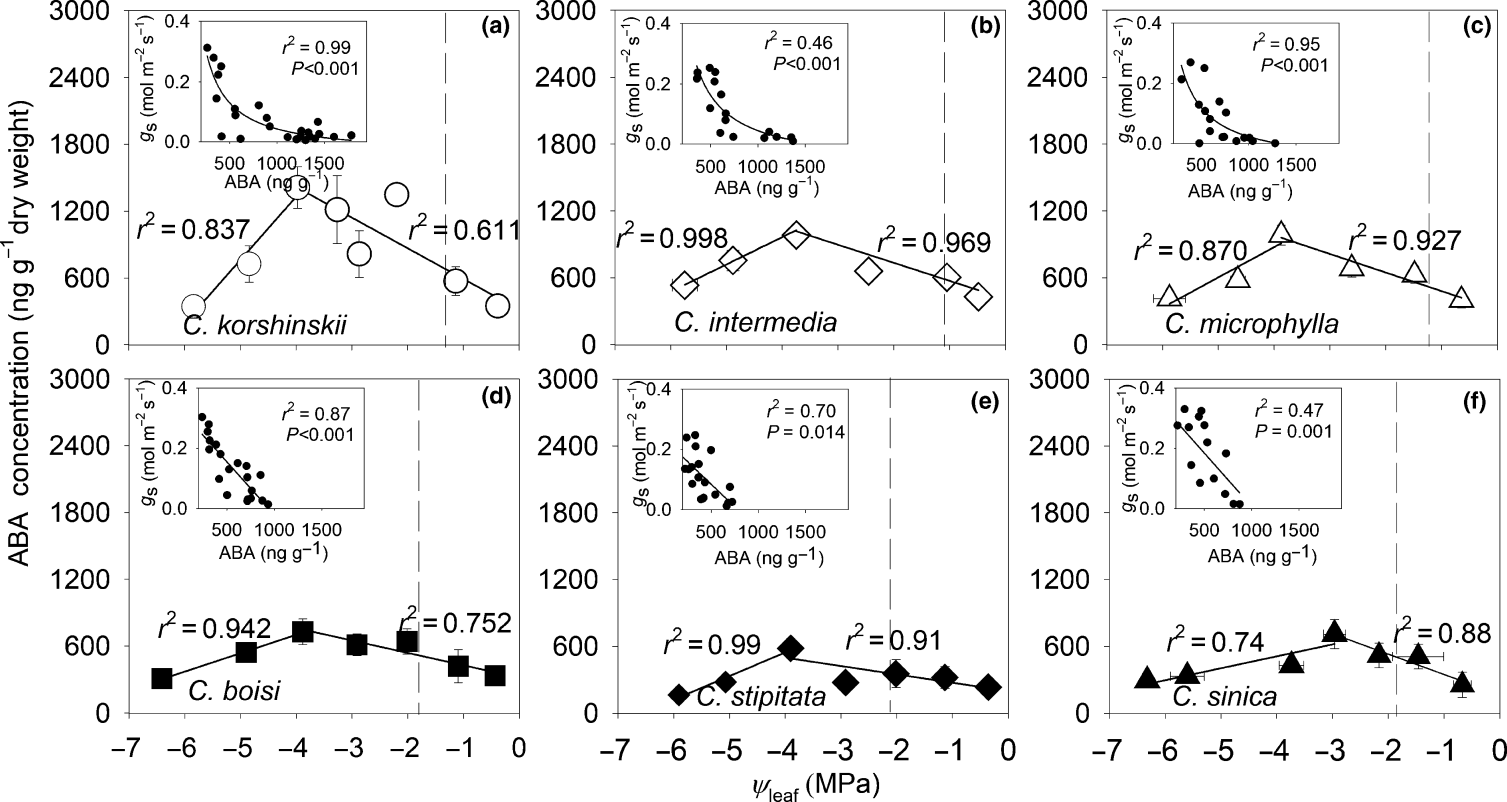
Relationship between leaf ABA concentration, leaf water potential and stomatal conductance. The relationship between leaf abscisic acid (ABA) concentration and predawn leaf water potential (Ψ_leaf_) in six *Caragana* species: (a) *C. korshinskii*, (b) *C. intermedia*, (c) *C. microphylla*, (d) *C. boisi*, (e) *C. stipitate* and (f) *C. sinica*. The inset panels show the relationship between stomatal conductance (*g*_s_) and leaf ABA concentration before ABA concentration decreased. The inset panels show the correlation coefficients (*r* ^2^) and probabilities (*P*) of the fitted inverse first order regressions (a–c) and the fitted linear regressions (d–f). The vertical dashed line represents Ψ_leaf_ at 50% loss of *g*_s_. The bars are ± 1 SE of the mean (*n* = 4) when larger than the symbol.

The leaf ABA concentration increased linearly until the predawn Ψ_leaf_ reached −3.0 to −4.0 MPa across species, and then decreased (Fig. 5). However, the peak leaf ABA concentration was 1000–1500 ng g^−1^ dry weight (DW) in species from low‐MAP environments, vs 550–750 ng g^−1^ DW in species from high‐MAP environments (*t*‐test, *P* = 0.02; Fig. 5; Table S2). Species showed a significant negative correlation between *g*_s_ and ABA concentration as Ψ_leaf_ decreased (Fig. 5 inset panels). The ABA concentration at *g*_s_ *P*_50_ only varied from 406 ng g^−1^ DW in *C. stipitata* to 670 ng g^−1^ DW in *C. korshinskii*, and no significant differences were observed between species of low‐ vs high‐MAP environments (*t*‐test; *P* = 0.111). Species from low‐MAP environments achieved higher ABA concentrations at higher values of Ψ_leaf_ than those from high‐MAP environments (Fig. 5) and *g*_s_ *P*_50_ was about −1.2 MPa in low‐MAP environments, whereas it was more negative in species from high‐MAP environments (about −1.9 MPa). Notably, the slopes of ABA accumulation with decreasing Ψ_leaf_ were not correlated with *K*_leaf_ *P*_50_ (*r* ^2^ = 0.007, *P* = 0.131).

### Optical embolisms

**Fig. 6:**
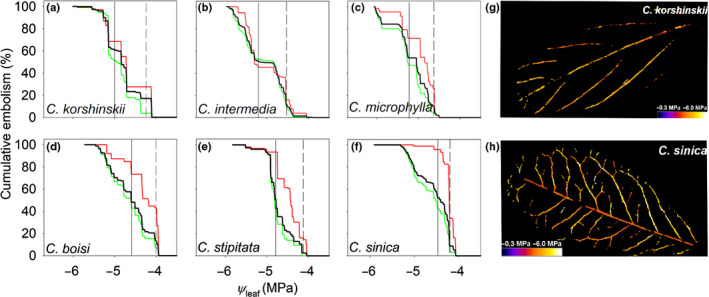
Cumulative embolism with decreasing leaf water potential (Ψ_leaf_) in 1° and 2° order veins. Leaf vein optical vulnerability curves of six *Caragana* species from (a–c) low mean annual precipitation (MAP) environments and (d–f) high‐MAP environments: (a) *C. korshinskii*, (b) *C. intermedia*, (c) *C. microphylla*, (d) *C. boisi*, (e) *C. stipitata* and (f) *C. sinica*. The cumulative embolism in 1° and 2° order veins (embolized pixels/total embolized pixels × 100%) is shown by the black line, in only the 1° order veins by the red line, and in only the 2° order veins by the green line. The vertical dashed and solid lines denote the Ψ_leaf_ at which leaf conduits reached 12% cumulative embolism (*PLC*_major_ *P*_12_) and 50% cumulative embolism (*PLC*_major_ *P*_50_) in 1° and 2° vein orders. The spatial distribution of embolisms are shown only for (g) *C. korshinskii* (from a low‐MAP environment) and (h) *C. sinica* (from a high‐MAP environment) (see Supporting Information Videos S1, S2).

Discrete embolisms were not observed propagating in the midrib until Ψ_leaf_ reached *c*. −4.0 MPa in all six species (Fig. 6; Videos S1, S2). Embolisms then propagated from the midrib to the 2° vein. *PLC*_major_ *P*_12_ and *PLC*_major_ *P*_50_ ranged from −4.0 to −4.6 MPa and from −4.5 to −5.2 MPa, respectively (Fig. 6). On average, *PLC*_major_ *P*_12_ and *PLC*_major_ *P*_50_ were slightly more negative in three species from low‐MAP environments than in three species from high‐MAP environments (*t*‐test, *P* = 0.011–0.025; Fig. S7). *PLC*_major_ *P*_12_ did not correlate with Ψ_leaf_ at ABA peak (*r* ^2^ = 0.004, *P* = 0.902), and neither *PLC*_major_ *P*_12_ nor *PLC*_major_ *P*_50_ significantly correlated with *K*_leaf‐max_ or *K*_leaf_ *P*_50_ across six *Caragana* species (Fig. S8).

### Variation in physiological sequences of events during dehydration

**Fig. 7:**
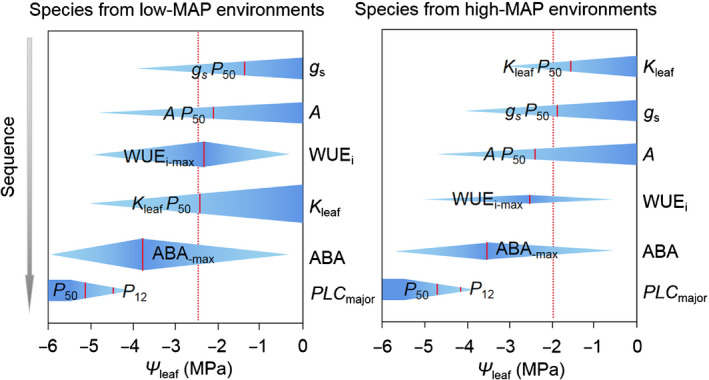
The sequence of physiological responses to leaf dehydration in *Caragana* from low and high mean annual precipitation environments. Changes in stomatal conductance (*g*_s_), net photosynthesis (*A*), intrinsic water‐use efficiency (WUE_i_), leaf hydraulic conductance (*K*_leaf_), leaf ABA concentration and cumulative embolism with decreasing leaf water potential (Ψ_leaf_) in *Caragana* species from low‐ and high‐mean annual precipitation environments are shown. The vertical short solid red lines in each response variable represent the species mean Ψ_leaf_ at 50% loss of *g*_s_ (*g*_s_ *P*_50_), Ψ_leaf_ at 50% loss of *A* (*A P*_50_), Ψ_leaf_ at maximum WUE_i_ (WUE_i‐max_), Ψ_leaf_ at 50% loss of *K*_leaf_ (*K*_leaf_ *P*_50_), Ψ_leaf_ at peak ABA concentration (ABA_‐max_), Ψ_leaf_ at which leaf conduits reached 12% cumulative embolism (*PLC*_major_ *P*_12_) and 50% cumulative embolism (*PLC*_major_ *P*_50_) in 1° and 2° vein orders. The vertical dotted red line represents the species mean turgor loss point (π_tlp_).

Species from low‐ vs high‐MAP environments displayed different sequences of physiological events during dehydration (Fig. 7) and thus differed in their hydraulic safety margins (Table S2). In species from low‐MAP environments, a higher sensitivity of stomatal conductance (*g*_s_) to decreasing leaf water potential, triggered by increases in ABA, helps to optimize WUE_i_ until the turgor loss point (Fig. 7). Species from high‐MAP environments still operated close to or below the turgor loss point, whereas species from low‐MAP environments had greater stomatal (*g*_s_ *P*_50_) and photosynthetic (*A P*_50_) sensitivity before the turgor loss point (*t*‐tests, *P* ≤ 0.002; Table S2). Species from high‐MAP environments displayed greater hydraulic safety (*K*_leaf_ *P*_50_) before turgor loss point (*t*‐test; *P = *0.02; Fig. 7; Table S2). However, no significant differences were found between stomatal and photosynthetic responses, with *g*_s_ *P*_50_ occurring on average across species 0.63 MPa before *A P*_50_ (Table S2). Across all species, embolism formation occurred only under severe and prolonged drought (Fig. 7).

## Discussion

### High leaf hydraulic efficiency and safety as a strategy to cope with aridity in *Caragana* species

Because of the important role that precipitation plays in shaping species’ distribution, in recent decades there has been a considerable interest in understanding the physiological adaptation of species to contrasting water availability (Pockman & Sperry, 2000; Maherali *et al*., 2004; Choat *et al*., 2012, 2007; Engelbrecht *et al*., 2007; Blackman *et al*., 2014). Common garden experiments have unraveled important correlations between hydraulic traits and environmental parameters from the species’ original habitat, suggesting a genetic basis to these physiological adaptations (Scoffoni *et al*., 2016; Jankowski *et al*., 2019; Ignazia *et al*., 2020). Here, we report that closely‐related species from habitats with varying MAP have diversified to exhibit both a high leaf hydraulic efficiency and safety under low water resource availability. Indeed, we found a strong significant positive correlation between water potential at 50% loss of leaf hydraulic conductance (*K*_leaf_ *P*_50_; i.e. hydraulic safety) and maximum leaf hydraulic conductance (*K*_leaf‐max_; i.e. hydraulic efficiency) across 10 *Caragana* species growing in a common garden. This high hydraulic safety and efficiency strategy would be particularly adaptive for species growing in arid environments: species can both resist drought‐induced hydraulic impairment and efficiently transport water when water is made available (i.e. after a rare rainfall event). Indeed, our results show a significant correlation between *K*_leaf_ *P*_50_ and original habitat MAP in these closely related species, with species from habitats with lower MAP exhibiting more negative *K*_leaf_ *P*_50_. These results are in accordance with data showing similar trends in a meta‐analysis and across diverse Australian species measured *in situ* studies (Blackman *et al*., 2012, 2014; Nardini & Luglio, 2014). Our results, combined with those that have been published previously, emphasize that *K*_leaf_ *P*_50_ is an important trait in shaping species’ distribution across water‐availability gradients; similar suggestions have been made for for *K*_stem_ *P*_50_ (Choat *et al*., 2012). Furthermore, *K*_leaf‐max_ was also correlated with original habitat MAP, suggesting that species native to low‐MAP environments exhibit higher *K*_leaf‐max_. High efficiency and safety would be particularly advantageous in *Caragana* growing in these cold and low‐MAP environments. Indeed, the accumulation of snow in winter results in high soil water availability in the spring after the snow melts, and along with spring rainfall, provides plants with favorable soil moisture at the beginning of the growing season. A high *K*_leaf‐max_ in these species would therefore help to keep stomata open during the day through changes in VPD (Scoffoni *et al*., 2018) and help the plant achieve a high photosynthetic rate during this short growing season to produce flowers and mature seeds before the severely dry summer that starts in July (Fang *et al*., 2017).

A positive trend between hydraulic safety and efficiency, such as that observed here across *Caragana* species, can be surprising at first glance given previous findings. A negative trend instead between hydraulic safety and efficiency has been reported in many studies. Previous studies showed closely‐related species did not possess a ‘safe’ stem hydraulic system (i.e. one that withstands a very negative *K*_stem_ *P*_50_) while being highly ‘efficient’ (i.e. a very high *K*_stem_) whereas no correlation was found in leaves (Chen *et al*., 2009; Hao *et al*., 2012). In meta‐analyses of diverse species, the trade‐off was observed in both leaves and stems, which pointed to a lack of data for species that would exhibit both extreme high hydraulic efficiencies and safety (Gleason *et al*., 2016; Scoffoni & Sack, 2017). In stems, lack of such a combination relates to limitations of the xylem, as characteristics of the pit membranes, lumen dimensions and the number of xylem conduits can impact the efficiency of water movement and safety from embolism (Wheeler *et al*., 2005; Sperry *et al*., 2006; Pittermann, 2010; Gleason *et al*., 2016; Pfautsch *et al*., 2016). In leaves, such a trade‐off might be driven by similar reasons to those for stems if the proportion of resistance in the leaf xylem pathways is higher than that in the outside‐xylem pathways. However, these two meta‐analyses pointed to the weakness of the trend, with species displaying strong variation in both maximum efficiencies and vulnerabilities of the hydraulic system (Gleason *et al*., 2016; Scoffoni & Sack, 2017). Thus, the strong variation in *K*_leaf‐max_ for species with *K*_leaf_ *P*_50_ values > −3 MPa indicates that there is room for species to achieve high hydraulic efficiency and safety in a given system, as we report here in *Caragana*. High hydraulic efficiency and safety has also been reported across four genotypes of coffee (Nardini *et al*., 2014). This positive trend could reflect a diversity of drought resistance traits evolved in both the xylem and outside‐xylem pathways (Scoffoni & Sack, 2017). Our results confirm that evolving a xylem resistant to embolism formation did not limit maximum hydraulic efficiency. Indeed, we found no correlation between *PLC*_major_ *P*_12_, *PLC*_major_ *P*_50_ and *K*_leaf‐max_ (*P* = 0.252–0.359).

Finally, we note that all our species are deciduous, thus eventually escaping the most intense/prolonged drought of the season. Such a life strategy could affect the hydraulic strategies described here. Indeed, previous studies have found that evergreen species tend to have lower maximum stem specific hydraulic conductance than deciduous ones, but more negative Ψ_leaf_ at 50% loss of stem hydraulic conductance (Choat *et al*., 2005; Kröber *et al*., 2014). However, at the leaf level this relationship remains unclear. Indeed, a recent meta‐analysis found that across 215 angiosperm woody species, evergreen species had more negative *K*_leaf_ *P*_50_ values than deciduous ones (Scoffoni & Sack, 2017). Our analysis of the 46 angiosperm deciduous and 84 evergreen deciduous species from Scoffoni & Sack (2017) showed no significant differences between *K*_leaf‐max_ (*t*‐test; *P* = 0.38), thus suggesting that the safety–efficiency trade‐off was not necessarily driven by differences in species life strategies. Furthermore, we note that a positive correlation between *K*_leaf‐max_ and *K*_leaf_ *P*_50_ was also found across four genotypes of coffee, an evergreen species (Nardini *et al*., 2014). Future work is needed to further explore the causes behind the trade‐off observed between *K*_leaf‐max_ and *K*_leaf_ *P*_50_ across diverse species (Scoffoni & Sack, 2017). We note that indeed, species with long‐lived leaves might require cells and xylem to be built to withstand greater tensions, which could impact to some extent maximum *K*_leaf_. Across our deciduous species, no significant differences in LMA (Table S3) were found, suggesting that mesophyll anatomy, including differences in cell sizes (and thus cell wall thickness) (John *et al*., 2013) and mesophyll cell layers, which are major drivers of LMA (John *et al*., 2017), might not vary much across our species. Indeed, variation in mesophyll anatomy has been shown to influence maximum *K*_leaf_ (Buckley, 2015). Thus, it remains unclear whether a combined high *K*_leaf_ and more negative *K*_leaf_ *P*_50_ could be observed across more diverse species, including both evergreen and deciduous species. Future work in leaves is needed to establish the underlying causes of these patterns.

### Drivers behind the high hydraulic safety and efficiency trend in *Caragana*

Our results point to leaf venation playing a role in driving higher *K*_leaf‐max_. Both minor and major VLA were tightly associated with *K*_leaf‐max_ (Fig. 4b,c). Indeed, studies have shown that an increase in VLA can help reduce the hydraulic resistance of both the xylem and outside‐xylem pathways by adding more routes for water to travel through, shortening the distance from the xylem to the site of evaporation in the leaf (Brodribb & Jordan, 2008; Scoffoni *et al*., 2016), increasing the bundle sheath surface area in the leaf (Caringella *et al*., 2015) and thus decreasing the resistance of water movement from the vein xylem to the mesophyll (Sack & Frole, 2006; Brodribb & Jordan, 2008). Furthermore, our data suggest that conductance through the midrib played a role in the increase in *K*_leaf‐max_ across species, as it was associated with midrib xylem area per leaflet area in species with higher values in low‐MAP environments (Fig. 4d). Finally, our data suggest that leaf capacitance at full turgor could also have helped increase *K*_leaf‐max_ in species from low‐MAP environments. Indeed, the leaf area specific capacitance at full turgor (C*_FT_) was closely correlated with *K*_leaf‐max_, with higher values observed in species from low‐MAP environments (Fig. 4a). The C*_FT_ contributes to the ability of the leaf to meet transpirational demand by minimizing transient fluctuations in mesophyll water potential (Sack *et al*., 2003; Xiong & Nadal, 2020).

Contrary to the idea that high *K*_leaf‐max_ is potentially achieved through diversification of both xylem and outside‐xylem tissues, the more negative *K*_leaf_ *P*_50_ displayed in species from low‐MAP environments appears to be driven mainly by changes in outside‐xylem pathways. Our results showed that across the six species, 66 ± 6.5% of the decrease in *K*_leaf_ occurred before the turgor loss point, and 94 ± 3.1% of the decrease in *K*_leaf_ occurred before initial embolism formation in leaf mid‐veins (at a Ψ_leaf_ close to about −4.0 MPa). The lack of embolism before turgor loss point has indeed been observed in most species tested (Scoffoni & Sack, 2017). Our results corroborate those of previous studies suggesting that *K*_ox_ was principally involved in the early decrease of *K*_leaf_ (Sack *et al*., 2016; Scoffoni & Sack, 2017; Scoffoni *et al*., 2017). The more negative *K*_leaf_ *P*_50_ could be achieved through changes in aquaporin expression, mesophyll and bundle sheath anatomy, cell wall composition and/or in changes in intercellular airspace distribution (Buckley *et al*., 2015; Earles *et al*., 2018; Ohtsuka *et al*., 2018; Scoffoni *et al*., 2018). While we did not quantify aquaporin expression or cross‐sectional anatomy, the tight correlation between *K*_leaf_ *P*_50_ and π_tlp_ suggests a link between outside‐xylem pathways and hydraulic vulnerability. Such a link has been observed across a diverse set of species (Scoffoni & Sack, 2017), and studies have suggested that a more negative π_tlp_ could help maintain cell integrity and hydraulic pathways outside the xylem under increased drought (Scoffoni *et al*., 2014) and extend the range of Ψ_leaf_ over which the leaf remains turgid, thereby maintaining leaf hydraulic function (Sack *et al*., 2003; Lenz *et al*., 2006). A more negative π_tlp_ could also allow aquaporins to remain activated longer during drought (Kim & Steudle, 2007; Scoffoni & Sack, 2017). Aquaporins could deactivate in response to a change in cell turgor, especially within the vascular parenchyma and/or bundle sheath (Kim & Steudle, 2007; Shatil‐Cohen *et al*., 2011; Pantin *et al*., 2013) which represent a large bottleneck in outside‐xylem pathways, as water needs to move past these cells to reach the mesophyll and stomata. In this study, we defined the leaf hydraulic safety as the leaf water potential (Ψ_leaf_) at 50% loss of *K*_leaf_ (*K*_leaf_ *P*_50_), as it has typically been defined throughout the leaf hydraulic literature (Brodribb & Holbrook, 2006; Scoffoni *et al*., 2017, 2011; Guyot *et al*., 2012; Bucci *et al*., 2013; Laur & Hacke, 2014). The term ‘safety’ originated from usage in stem hydraulics as safety against embolism formation which has been shown to be a major cause of plant mortality world‐wide (Choat *et al*., 2012). Notably, across our species set, no embolism occurred at *K*_leaf_ *P*_50_. We suggest that the safety–efficiency trade‐off in leaves might be better referred to as an efficiency–sensitivity trade‐off. Similarly, leaf hydraulic (or stomatal or photosynthetic) sensitivity (instead of vulnerability) curves might better reflect the leaf‐level physiological response to dehydration if embolisms are not involved.

### Hydraulics and gas exchange

We found no association between *K*_leaf‐max_ and *g*_s‐max_ or *A*_max_ across 10 *Caragana* species. These results are contrary to previous findings of a strong relationship between *K*_leaf‐max_ and gas exchange in both closely‐related and diverse angiosperms (Brodribb *et al*., 2005; Scoffoni *et al*., 2016), pointing to a hydraulic basis for the evolution of high photosynthetic rates and thus growth (Scoffoni *et al*., 2016). However, across nine C_4_ grasses, a lack of correlation between *K*_leaf‐max_ and either stomatal conductance or photosynthesis was also observed (Ocheltree *et al*., 2016). The lack of association observed across *Caragana* species does not necessarily indicate the absence of a role for leaf hydraulics in increasing photosynthetic rates across species. Indeed, species did not vary in *g*_s‐max_ or *A*_max_, but strongly varied in *K*_leaf‐max_, suggesting that some species display greater hydraulic supply than is needed for photosynthesis. Indeed, species varied in their hydraulic supply to demand ratio (*K*_leaf‐max_/*g*_s‐max_), with species from low‐MAP environments displaying greater values. Further, a negative correlation was observed between *K*_leaf‐max_/*g*_s‐max_ and habitat MAP (Fig. 2a). Thus, our results suggest that the increase in *K*_leaf‐max_ in species from low‐MAP environments might be beneficial to species survival for four reasons: first, it helps mitigate the greater evaporative loads induced by high vapour pressure deficits in dry environments (Brodribb *et al*., 2007; Scoffoni *et al*., 2016, 2011); second, it reduces leaf temperature through evaporative cooling; third, it helps reduce xylem tension (and thus embolisms) at any given rate of transpiration; and fourth, it allows faster recovery of wilted tissues with efficient water transport after an occasional rainfall event post drought.

*Caragana* species from low‐ vs high‐MAP environments differed in their sequence of physiological responses to dehydration (Fig. 7). Species from low‐MAP environments were characterized by early stomatal closure (Fig. S2) and decrease in photosynthesis (Fig. S5) before hydraulic decrease (Fig. S1). Such high stomatal sensitivity appears to be associated with steep increases in foliar ABA concentration (Fig. 5) in *Caragana* species from low‐MAP environments compared with species from high‐MAP environments in the early stages of drought. Indeed, the ABA concentration at *g*_s_ *P*_50_ across species did not differ significantly between species from low‐ vs high‐MAP environments, but this value was achieved at significantly higher water potentials in species from low‐MAP environments (Fig. 5). This greater stomatal sensitivity, driven by an increase in ABA, contributed to optimizing the leaf WUE_i_ in species from low‐MAP environments (Negin & Moshelion, 2016) which enables these species to maintain carbon gain during dehydration (as net assimilation does not decrease as much as *g*_s_ with dehydration) while saving water (Fig. 3). Notably, the lack of correlation between the slopes of ABA accumulation with decreasing Ψ_leaf_ with *K*_leaf_ *P*_50_ implies that ABA did not have a significant role in driving changes in *K*_leaf_ during dehydration. Previous studies had indeed found that ABA could trigger aquaporin deactivation in bundle sheath cells, thus decreasing outside‐xylem hydraulic conductance (Shatil‐Cohen *et al*., 2011; Prado *et al*., 2013). Instead, *K*_leaf_ was less sensitive to dehydration in species from low‐MAP environments, enabling greater cell hydration during dehydration. Under more severe drought conditions, a marked decrease in ABA levels (Fig. 5) indicates a shift from ABA‐driven stomatal closure to water potential driven stomatal closure (Brodribb & McAdam, 2013), and the onset of leaf xylem embolism appears at around those water potential values (Fig. 7). By contrast, rapidly declining water potentials generated by the high *K*_leaf_ sensitivity to dehydration in species from high‐MAP environments would help contribute to stomatal closure (Scoffoni *et al*., 2018), as ABA levels were significantly lower in those species, and in turn help optimize WUEi during dehydration. We note that in low‐MAP environments, ABA was more efficient in optimizing WUE_i_ than *K*_leaf_ sensitivity to dehydration, as WUE_i_ was higher in species from these habitats, allowing for greater functionality under drier conditions. These results corroborate past studies reporting species exhibiting greater WUE in response to either simulated reduction in precipitation (Grossiord *et al*., 2017), or to different growth environments (Cornwell *et al*., 2010). Indeed, *Metrosideros polymorpha* growing in low‐MAP environments (< 400 mm yr^–1^) exhibits higher water use efficiency than when grown in high‐MAP environments (> 10 000 mm yr^–1^) (Cornwell *et al*., 2010).

### Limitations of a common garden approach

We tested leaf hydraulic efficiency and safety in *Caragana* species in a common garden environment. This approach is useful in exposing potential genotypic differences and has been shown to be essential in establishing evolutionary coordination of traits (Mason & Donovan, 2015; Scoffoni *et al*., 2016); however, it cannot fully reflect species’ responses to drought *in situ* and/or in plants grown under different water regimes. Here, the results from the common garden showed strong genotypic differences in leaf hydraulics between species from low‐ vs high‐MAP environments. Notably, we argue that these differences would be more pronounced *in situ*, or in plants grown under low water availability. Indeed, differences in leaf ontogeny for species grown *in situ* (or under low water availability) would most likely only reinforce these differences, as studies have found that species growing in arid habitats tend to have smaller leaves with higher major vein length per area, higher minor vein length per area, greater resistance to embolism formation and more negative turgor loss point (Bartlett *et al*., 2012; Sack & Scoffoni, 2013; Scoffoni & Sack, 2017). These anatomical differences would likely increase *K*_max_ and make *K*_leaf_ *P*_50_ more negative. Notably, the low variation in the leaf xylem hydraulic conductance measured with the optical method across species could reflect the limitations of having grown these species in a common garden, and the differences in *PLC*_major_ *P*_12_ and *PLC*_major_ *P*_50_ between species from low‐ and high‐MAP environments might be more pronounced *in situ*. Future studies should more closely examine the relationship between hydraulic traits *in situ* or when grown under contrasting water availability to unravel the maximum potential hydraulic response to drought.

### Conclusion

Across 10 *Caragana* species from a precipitation gradient of 110 mm to 1400 mm grown in a common garden, high hydraulic safety was positively related to high efficiency, with species from drier habitats exhibiting higher safety and efficiency, more negative π_tlp_ and higher area‐based C*_FT_, vein density and midrib xylem area per leaflet area. The higher *K*_leaf‐max_ in species from low‐MAP environments did not translate to higher *g*_s‐max_, and tighter stomatal control mediated by higher ABA accumulation during dehydration resulted in a rapid decrease in *g*_s_, thereby increasing hydraulic safety and water use efficiency. The results help advance our understanding of hydraulic performance in species from low‐MAP environments – a greater leaf hydraulic efficiency and safety, along with high water use efficiency, could allow species to grow and survive in arid and semiarid areas that are predicted to become drier and warmer with climate change.

## Author contributions

CS, XWF, NCT and FML designed the experiments and wrote the manuscript; XWF performed data analysis; CS, XWF and TPG prepared the figures; GQY and ZFN conducted most of the experiments. G‐QY and Z‐FN contributed equally to this work.

## Supporting information


**Fig. S1** Response of leaf hydraulic conductance to dehydration in 10 *Caragana* species.
**Fig. S2** Response of stomatal conductance to dehydration in six *Caragana* species.
**Fig. S3** Relationship between maximum stomatal conductance and mean annual precipitation and maximum leaf hydraulic conductance in 10 *Caragana* species.
**Fig. S4** Relationship between leaf water potential at 50% loss of stomatal conductance and mean annual precipitation in six *Caragana* species.
**Fig. S5** Response of leaf photosynthesis to dehydration in six *Caragana* species.
**Fig. S6** Variation in leaf venation architecture across 10 *Caragana* species.
**Fig. S7** Variation in leaf water potential at which leaf conduits reached 12% and 50% cumulative embolism in 1° and 2° vein orders in six *Caragana* species.
**Fig. S8** Relationships between leaf hydraulics and embolism resistance across six *Caragana* species.
**Methods S1** Additional materials and methods.
**Table S1** The ploidy, location and environment at the collection sites of the 10 *Caragana* species.

**Table S2** Summary of 37 morphological, anatomical and physiological traits and results of analysis of variance for the difference across species (one‐way ANOVAs) and between species from low‐ vs high‐rainfall environments (*t*‐test).
**Table S3** Leaf mass per unit area and water relations characteristics of the 10 *Caragana* species used in this study.

**Video S1** Progression of embolism in *Caragana* *korshniskii* (animated version of Fig. 6g).

**Video S2** Progression of embolism in *Caragana* *sinica* (animated version of Fig. 6h).
Please note: Wiley Blackwell are not responsible for the content or functionality of any Supporting Information supplied by the authors. Any queries (other than missing material) should be directed to the *New Phytologist* Central Office.
