# Supplementary material for: Combined high leaf hydraulic safety and efficiency provides drought tolerance in Caragana species adapted to low mean annual precipitation
Source: New Phytol. 2020 Sep 7;229(1):230–44. doi: 10.1111/nph.16845 (PMC7754512; doi:10.1111/nph.16845)
Supplement: Supplementary file 1 — Fig. S1 Response of leaf hydraulic conductance to dehydration in 10 Caragana species. Fig. S2 Response of stomatal conductance to dehydration in six Caragana species. Fig. S3 Relationship between maximum stomatal conductance and mean annual precipitation and maximum leaf hydraulic conductance in 10 Caragana species. Fig. S4 Relationship between leaf water potential at 50% loss of stomatal conductance and mean annual precipitation in six Caragana species. Fig. S5 Response of leaf photosynthesis to dehydration in six Caragana species. Fig. S6 Variation in leaf venation architecture across 10 Caragana species. Fig. S7 Variation in leaf water potential at which leaf conduits reached 12% and 50% cumulative embolism in 1° and 2° vein orders in six Caragana species. Fig. S8 Relationships between leaf hydraulics and embolism resistance across six Caragana species. Methods S1 Additional materials and methods. Table S1 The ploidy, location and environment at the collection sites of the 10 Caragana species. [file NPH-229-230-s001.pdf]

New Phytologist

***New Phytologist* Supporting Information**

Article title: **Combined high leaf hydraulic safety and efficiency provides drought tolerance in *Caragana* species adapted to low mean annual precipitation**

Authors: Guang-Qian Yao, Zheng-Fei Nie, Neil C. Turner, Feng-Min Li, Tian-Peng Gao, Xiang-Wen Fang and Christine Scoffoni

Article acceptance date: 22 July 2020

The following Supporting Information is available for this article:

Fig. S1 Response of leaf hydraulic conductance to dehydration in ten *Caragana* species.

Fig. S2 Response of stomatal conductance to dehydration in six *Caragana* species.

Fig. S3 Relationship between maximum stomatal conductance and (a) mean annual precipitation and (b) maximum leaf hydraulic conductance in ten *Caragana* species.

Fig. S4 Relationship between leaf water potential at 50% loss of stomatal conductance and mean annual precipitation in six *Caragana* species.

Fig. S5 Response of leaf photosynthesis to dehydration in six *Caragana* species.

Fig. S6 Variation in leaf venation architecture across ten *Caragana* species.

Fig. S7 Variation in leaf water potential at which leaf conduits reached 12% and 50% cumulative embolism in $1^o + 2^o$ vein orders in six *Caragana* species.

Fig. S8 Relationships between leaf hydraulics and embolism resistance across six *Caragana* species.

Table S1 The ploidy, location and environment at the collection sites of the ten *Caragana* species.

Table S2 Summary of 37 morphological, anatomical and physiological traits and results of analysis of variance for the difference across species (one-way ANOVAs) and between species from low- *vs*. high-rainfall environments (*t*-test).

Table S3 Leaf mass per unit area and water relations characteristics of the ten *Caragana* species used in this study.

Methods S1 Additional Materials and Methods.

Video S1 Progression of embolism in *C. korshniskii*. Animated version of Fig 6g.

Video S2 Progression of embolism in *C. sinica*. Animated version of Fig 6h.

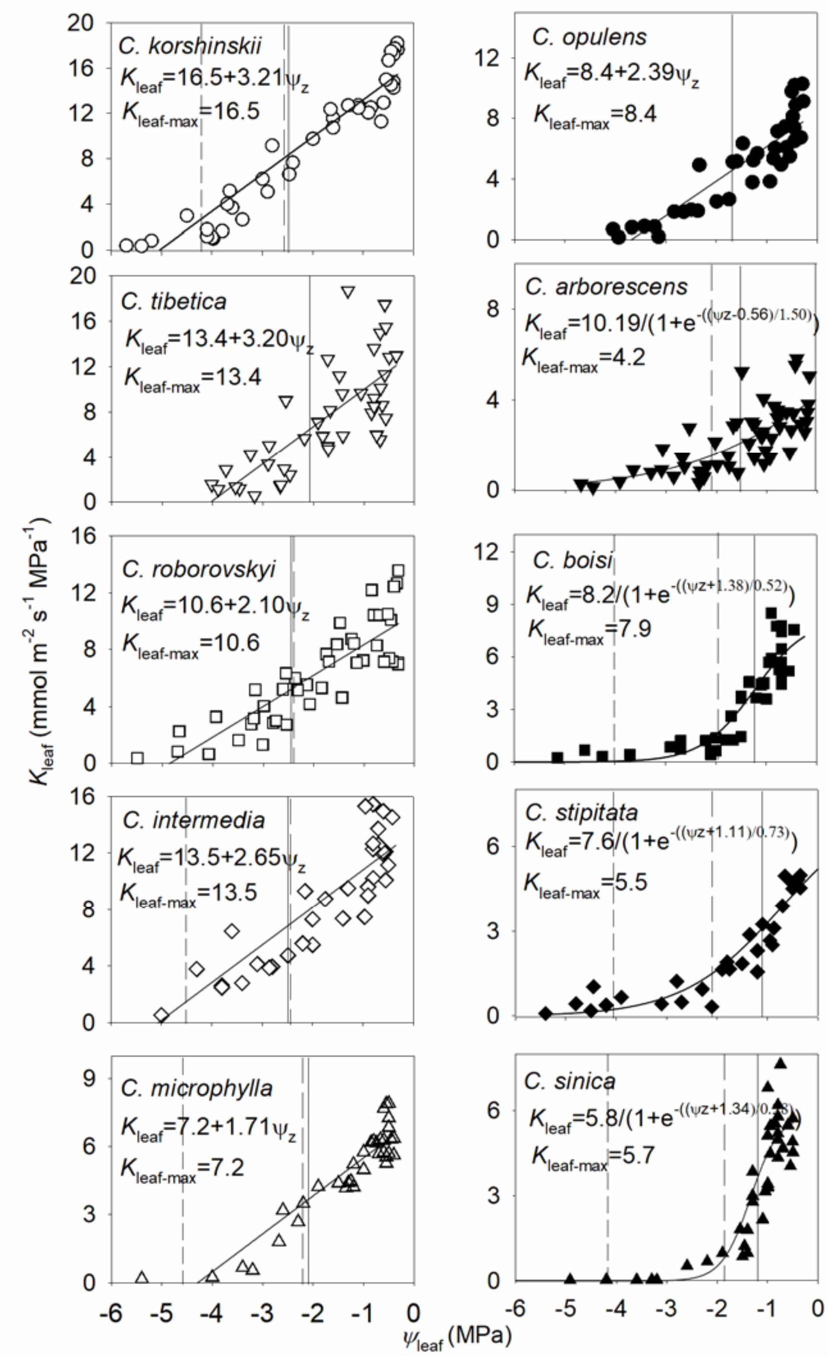

Fig. S1 Response of leaf hydraulic conductance to dehydration in ten *Caragana* species. The relationship between leaf hydraulic conductance (K_{leaf}) and leaf water potential (Ψ_{leaf}) in five *Caragana* species from low mean annual precipitation (MAP) environments (open symbols) in the left panels and five species from high-MAP environments (closed symbols) in the right panels, ordered from top to bottom in each column from lowest to highest MAP in their native

habitat. The vertical solid line represents Ψ_{leaf} at 50% loss of leaf hydraulic conductance (K_{leaf} P_{50}). The vertical long dashed line represents the turgor loss point (π_{tlp}) measured by the pressure-volume method, and the vertical short dashed line represents the onset of leaf xylem embolism [the Ψ_{leaf} at which leaf conduits reached 12% cumulative embolism (PLC_{major} P_{12}) in 1° + 2° vein orders]. The equation in each panel is of the best-fit function chosen from maximum likelihood between a linear, a three-parameter sigmoidal, a logistic and an exponential function (see the main text *Materials and Methods* section). Maximum values for K_{leaf} ($K_{\text{leaf-max}}$) are given for each species.

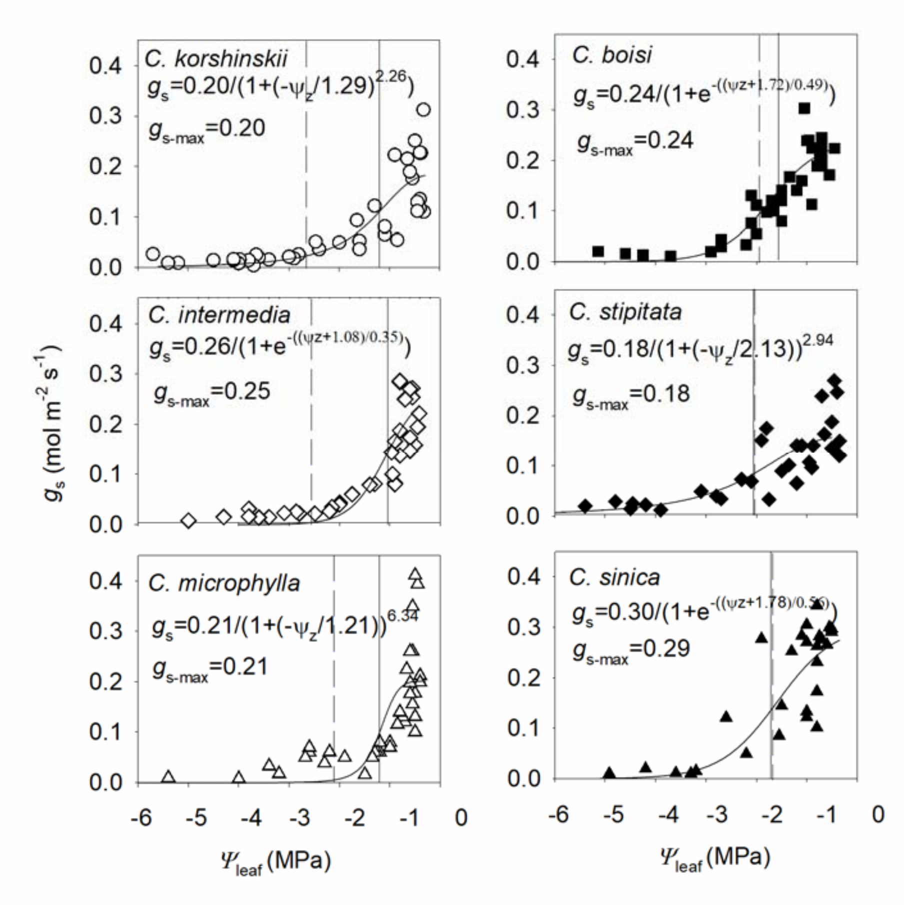

Fig. S2 Response of stomatal conductance to dehydration in six *Caragana* species. The relationship between stomatal conductance (g_s) and predawn leaf water potential (Ψ_{leaf}) in three *Caragana* species from low mean annual precipitation (MAP) environments (open symbols) in the left panels and three species from high-MAP environments (closed symbols) in the right panels, ordered from top to bottom in each column from lowest to highest MAP in their native habitat. The vertical solid line represents Ψ_{leaf} at 50% loss of g_s (g_s P_{50}), and the vertical long dashed line represents the turgor loss point (π_{tlp}) measured by the pressure-volume method. The equation in each panel is of the best-fit function chosen from maximum likelihood between a linear, a three-parameter sigmoidal, a logistic and an exponential function (see the main text *Materials and Methods* section). Maximum values for g_s ($g_{s\text{-}max}$) are given for each species.

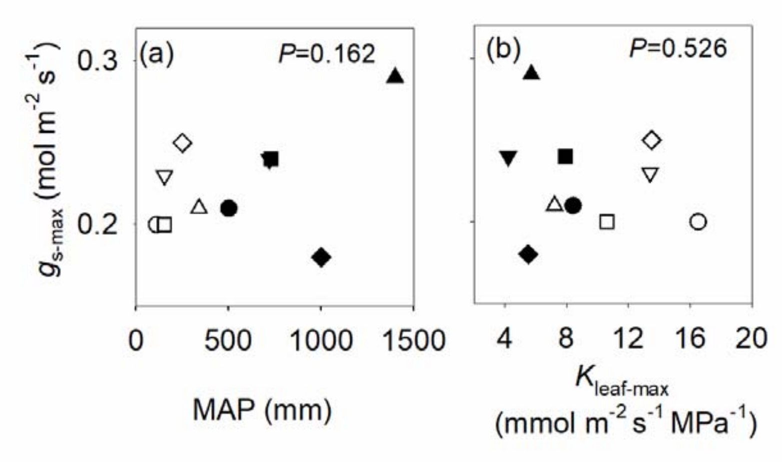

Fig. S3 Relationship between maximum stomatal conductance and (a) mean annual precipitation and (b) maximum leaf hydraulic conductance in ten *Caragana* species. The relationship between maximum stomatal conductance ($g_{s\text{-max}}$) and (a) mean annual precipitation (MAP), and (b) maximum leaf hydraulic conductance ($K_{leaf\text{-max}}$) in three *Caragana* species from low-MAP environments (open symbols) and three species from high-MAP environments (closed symbols) in their native habitat. *C. korshinskii*, ○ ; *C. tibetica*,▽; *C. roborovskyi*, □ ; *C. intermedia*, ◇ ; *C. microphylla*, △ ; *C. opulens*, ●; *C. arborescens*, ▼; *C. boisi*, ■; *C. stipitata*, ◆; and *C. sinica*, ▲.

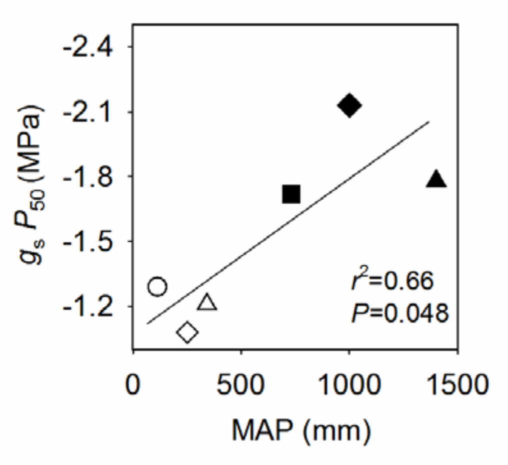

Fig. S4 Relationship between leaf water potential at 50% loss of stomatal conductance and mean annual precipitation in six *Caragana* species. The relationship between the predawn leaf water potential at 50% loss of stomatal conductance (g_s P_{50}) and mean annual precipitation (MAP) among six *Caragana* species, three from low-MAP environments (open symbols) and three from high-MAP environments (closed symbols) in their native habitat. The correlation coefficient (r^2) and probability (P) of the linear regression is given. *C. korshinskii*, ○; *C. intermedia*, ◇ ; *C. microphylla*, △ ; *C. boisi*, ■; *C. stipitata*, ◆; and *C. sinica*, ▲.

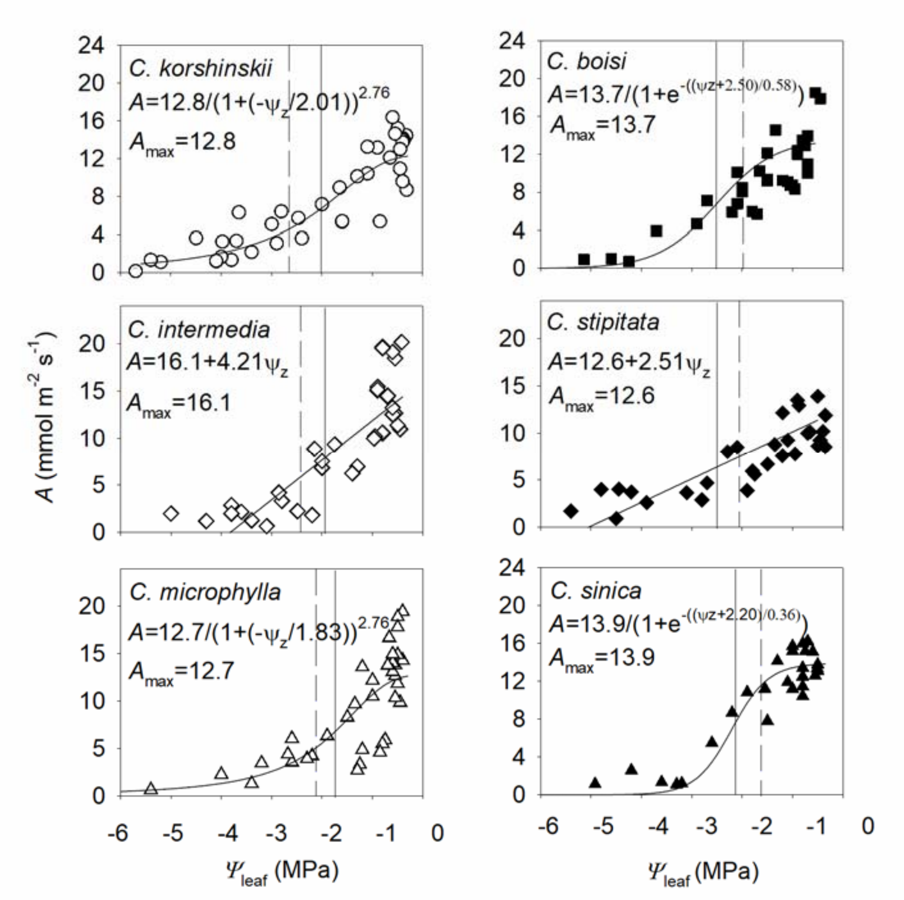

Fig. S5 Response of leaf photosynthesis to dehydration in six *Caragana* species. The relationship between net photosynthetic rate (A) with predawn leaf water potential (Ψ_{leaf}) in three *Caragana* species from low mean annual precipitation (MAP) environments (open symbols) in the left panels and three species from high-MAP environments (closed symbols) in the right panels, ordered from top to bottom in each column from lowest to highest MAP in their native habitat. The vertical solid line represents Ψ_{leaf} at 50% loss of A ($A\ P_{50}$), and the vertical long dashed line represents the turgor loss point (π_{tlp}) measured by the pressure-volume method. The equation in each panel is of the best-fit function chosen from maximum likelihood between a linear, a three-parameter sigmoidal, a logistic and an exponential function (see the main text *Materials and Methods* section). Maximum values for A (A_{max}) are given for each species.

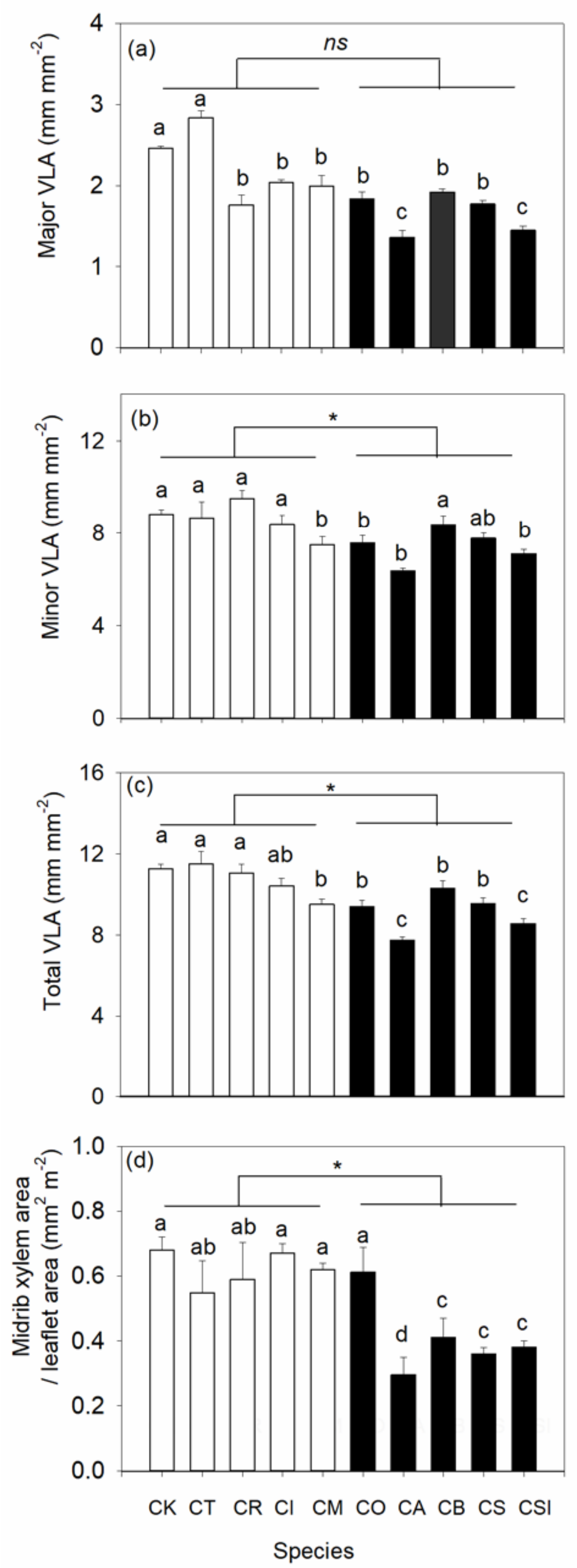

(a)
ns
Major VLA (mm mm-2)
(b)
*
Minor VLA (mm mm-2)
(c)
*
Total VLA (mm mm-2)
(d)
*
Midrib xylem area / leaflet area (mm2 m-2)
CK CT CR CI CM CO CA CB CS CSI
Species

Fig. S6 Variation in leaf venation architecture across ten *Caragana* species. (a) The major vein length per area (VLA), (b) minor VLA, (c) total VLA, and (d) midrib xylem area per leaflet area for ten *Caragana* species ordered left to right from lowest to highest mean annual precipitation (MAP) in their native habitat. The major vein density is the sum of the densities of the 1°, 2°, 3° order veins; minor vein density is the sum of the densities of the 4° and higher order veins, and total VLA is the sum of the major and minor VLA. Values are the means + one standard error of the mean (n= 6) and the different letters indicate significant differences between the means (one-way ANOVA followed by a post-hoc Duncan test; P <0.05). Asterisks indicate significant differences (t-test, * P<0.05) and *ns* indicate no significant difference (t-test, *ns* P>0.05) between the means of species from low- *vs.* high-MAP environments. White bars represent species from low-MAP environments, and black bars represent species from high-MAP environments. CK, *C. korshinskii*; CT, *C. tibetica*; CR, *C. roborovskyi*; CI, *C. intermedia*; CM, *C. microphylla*; CO, *C. opulens*; CA, *C. arborescens*; CB, *C. boisi*; CS, *C. stipitata*; CSI, *C. sinica*.

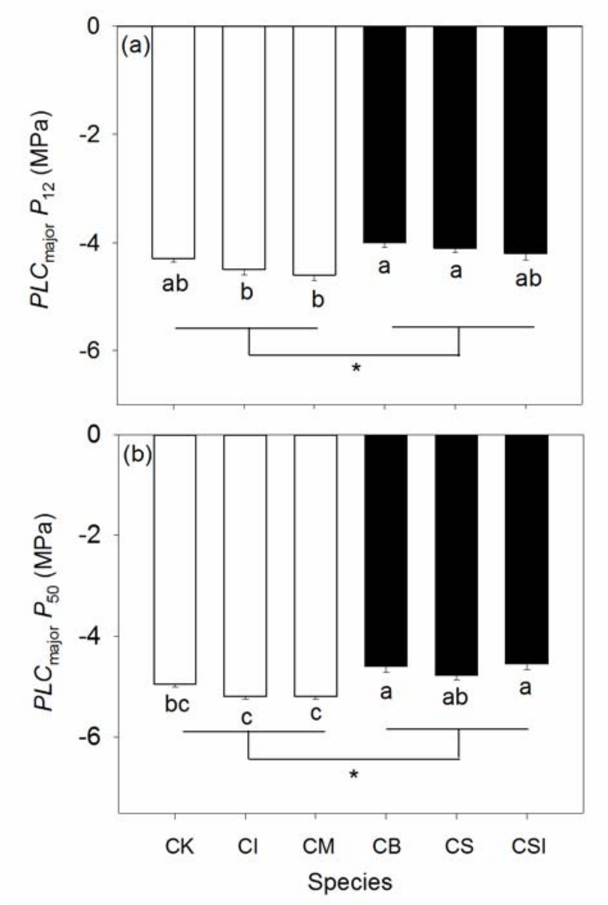

Fig. S7 Variation in leaf water potential at which leaf conduits reached 12% and 50% cumulative embolism in 1º + 2º vein orders in six *Caragana* species. The leaf water potential (Ψ_{leaf}) at which leaf conduits reached 12% cumulative embolism (PLC_{major} P_{12}) and 50% cumulative embolism (PLC_{major} P_{50}) in 1º + 2º vein orders in three *Caragana* species from low mean annual precipitation (MAP) environments (white bars) and three species from high-MAP environments (black bars) ordered from left to right from lowest to highest MAP in their native habitat. Values are the means - one standard error of the mean (n=3), the different letters indicate significant differences between the means across species (one-way ANOVA followed by a post-hoc Duncan test; P<0.05), and the asterisk indicates significant differences between the means of species from low- *vs.* high-MAP environments (t-test, * P<0.05). CK, *C. korshinskii*; CI, *C. intermedia*; CM, *C. microphylla*; CB, *C. boisi*; CS, *C. stipitata*; CSI, *C. sinica*.

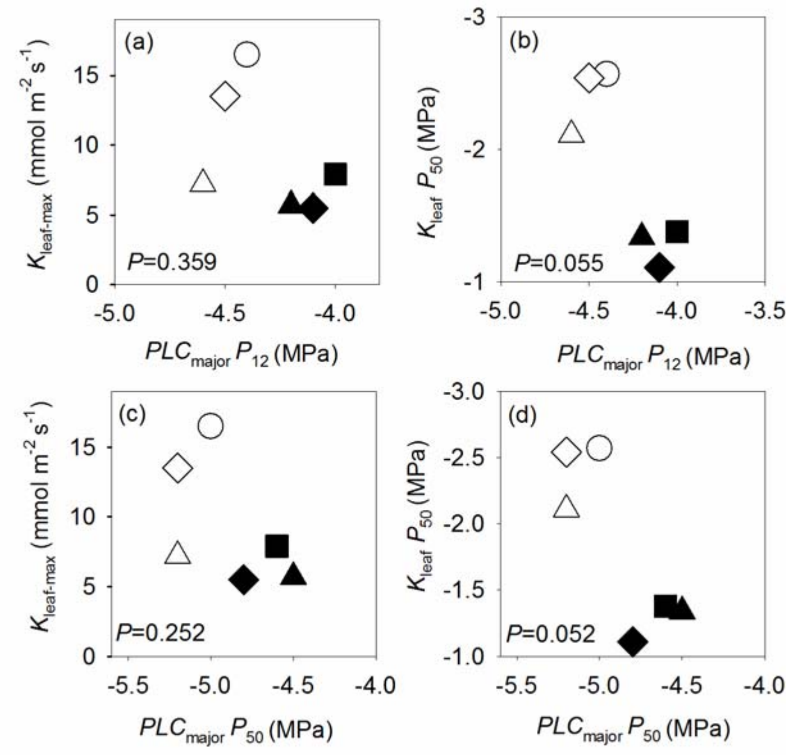

Fig. S8 Relationships between leaf hydraulics and embolism resistance across six *Caragana* species. The relationship between maximum leaf hydraulic conductance ($K_{leaf-max}$) (a, c) and leaf water potential (Ψ_{leaf}) at 50% loss of leaf hydraulic conductance ($K_{leaf}\ P_{50}$) (b, d) and the Ψ_{leaf} at which leaf conduits reached 12% cumulative embolism ($PLC_{major}\ P_{12}$) and 50% cumulative embolism ($PLC_{major}\ P_{50}$) in 1° + 2° vein orders in three *Caragana* species from low mean annual precipitation (MAP) environments (open symbols), and three species from high-MAP environments (closed symbols). *C. korshinskii*, ○; *C. intermedia*, ◇ ; *C. microphylla*, △ ; *C. boisi*, ■; *C. stipitata*, ◆; and *C. sinica*, ▲.

Table S1 The ploidy, location and environment at the collection sites of the ten *Caragana* species.

Species	Ploidy	Region of collection	Habitat	Light regime	Altitude (m)	MAP (mm)	AI	MAT (°C)
C. korshinskii	2X	Alasha, Inner Mongolia	Desert and semi-desert	Canopy	1430	110	32.2	7.3
C. tibetica	2X	Alasha, Inner Mongolia	Desert and semi-desert	Canopy	1400	153	25.6	7.3
C. roborovskyi	2X	Alasha, Inner Mongolia	Desert and semi-desert	Canopy	1400	153	25.6	7.3
C. intermedia	2X	Damao, Inner Mongolia	Desert steppe	Canopy	1370	250	10.3	4.2
C. microphylla	2X	Xiwu, Inner Mongolia	Steppe	Canopy	995	340	5.41	2.6
C. opulens	2X	Ninxia, Gansu Province	Deciduous forest	Gaps, Forest edges	1900	500	3.12	6.3
C. arborescens	2X	Yingkou, Liaoning Province	Deciduous forest	Gaps, Forest edges	200	720	2.56	9.0
C. boisi	2X	Lixian, Sichuan Province	Deciduous forest	Gaps, Forest edges	1600	730	1.96	8.3
C. stipitata	2X	Hua Mountains, Shanxi Province	Deciduous forest	Gaps, Forest edges	1610	1000	1.47	6.1
C. sinica	3X	Tianmu Mountains, Zhejiang Province	Deciduous forest	Forest edges, under story	1450	1400	0.68	8.5

MAP, mean annual precipitation; AI, aridity index (potential evapotranspiration/MAP); MAT, mean annual temperature; MAP and MAT are averaged from 1971 to 2000 (data after 2000 not supplied) from the China Meteorological Data Sharing Service System.

Table S3 Leaf mass per unit area and water relations characteristics of the ten *Caragana* species used in this study.

Species	LMA (g m^{-2})	π_o (MPa)	π_{tlp} (MPa)	ε (MPa)	RWC_{TLP} (%)	SWC (g g^{-1})	C^*_{FT} (mmol m^{-2} MPa^{-1})
C. korshinskii	89.6±4.67**a**	-2.25±0.09**a**	-2.66±0.11**a**	15.1±1.10**a**	84.7±0.8**c**	3.15±0.072**a**	0.69±0.06**a**
C. tibetica	88.9±1.14**a**	-	-	-	-	-	-
C. roborovskyi	90.2±3.26**a**	-1.95±0.17**b**	-2.39±0.10**b**	16.1±0.98**a**	94.0±0.8**a**	3.06±0.078**abc**	0.70±0.03**a**
C. intermedia	90.0±5.73**a**	-1.85±0.07**b**	-2.42±0.22**b**	14.7±1.33**a**	80.5±0.5**d**	2.99±0.082**abc**	0.71±0.03**a**
C. microphylla	89.7±4.00**a**	-1.83±0.12**b**	-2.22±0.15**bc**	16.6±1.55**a**	85.1±1.1**c**	3.07±0.110**abc**	0.64±0.10**ab**
C. opulens	86.6±1.05**a**	-	-	-	-	-	-
C. arborescens	87.4±3.96**a**	-1.45±0.07**b**	-2.10±0.12**bc**	15.1±0.77**a**	68.5±8.2**e**	2.90±0.181**abc**	0.51±0.07**bc**
C. boisi	91.1±3.38**a**	-1.58±0.09**d**	-1.98±0.05**d**	18.6±2.03**a**	89.2±0.6**b**	2.85±0.133**abc**	0.51±0.04**ab**
C. stipitata	88.8±4.93**a**	-1.67±0.11**c**	-2.05±0.21**c**	17.5±0.84**a**	91.2±0.9**a**	2.69±0.024**bc**	0.50±0.07**ab**
C. sinica	101.4±1.71**a**	-1.51±0.06**d**	-1.77±0.14**d**	18.8±1.51**a**	90.2±0.5**ab**	2.65±0.095**c**	0.44±0.04**b**
Low MAP	89.7±0.26	-1.97±0.096	-2.42±0.091	15.6±0.44	86.1±2.83	3.06±0.32	0.685±0.015
High MAP	91.7±2.68	-1.55±0.047	-1.97±0.072	17.5±0.85	84.7±5.44	2.77±0.60	0.490±0.016
t-test	0.623	0.008	0.008	0.098	0.839	0.005	<0.001

LMA, leaf mass per area; π_o, osmotic pressure at full turgor; π_{tlp}, osmotic pressure at the turgor loss point; ε, modulus of elasticity; RWC_{TLP}, relative water content at turgor loss point; SWC, the saturated water content per dry mass; C^*_{FT}, area-based leaf-specific capacity at full turgor (mean±se, n=6). The values across species in each column were analyzed by analysis of variance (one-way ANOVA) and the different letters indicate significant differences between the means ($P < 0.05$), and the values of species from low mean annual precipitation (MAP) environments (*C. korshinskii*, *C. tibetica*, *C. roborovskyi*, *C. intermedia*, *C. microphylla*) *vs.* high-MAP environments (*C. opulens*, *C. arborescens*, *C. boisi*, *C. stipitata*, *C. sinica*) were analyzed by *t*-test.

Methods S1 **Additional Materials and Methods**

Seed germination and seedling management

In June to August 2012, seeds of nine species of *Caragana*: *C. korshinskii*, *C. tibetica*, *C. roborovskyi*, *C. intermedia*, *C. microphylla*, *C. opulens*, *C. arborescens*, *C. boisi*, and *C. stipitata* growing in regions varying in mean annual precipitation (MAP) from 110 mm to 1400 mm and varying in aridity index (AI=potential evapotranspiration/MAP) from 0.68 to 32.2 (Table S1) were collected from wild populations at physiological maturity when the pods change color from green to brown. After collection, ripe fruits were spread out in the laboratory at 20 °C until they opened, and the seeds were removed. The seeds were stored at 4.0 °C until the start of the experiment. In February 2013, seeds of the nine species were placed in Petri dishes lined with wet filter paper to germinate at 20 °C. After germination and when the root was ~0.3 mm long, three seedlings were transferred into each of 900 plastic pots (100 pots for each species), 270 mm high and 170 mm in diameter, containing 3.6 kg of a 1:1 (v:v) mixture of sieved peat soil and Perlite with a dry bulk density of 0.63 g cm^{-3} and a pot capacity (PC) of 35% after being saturated with water and allowed to drain for 48 h. *C. sinica* has no seeds and tissue was cultured from more than 100 individuals as described by Song *et al.* (2007).

Thirty-one days after sowing, the seedlings were thinned to one per pot and tissue-cultured seedlings of *C. sinica* were moved to the pots (one individual per pot, 100 pots). All pots were irrigated every 2 days to maintain the soil above 80% PC by weighing the pots and replacing the water lost.

Optical transmission

Briefly, a branch was cut off from the plant under water in the evening and left to rehydrate overnight. When Ψ_{leaf} reached values above -0.3 MPa, a single leaflet was placed under a stereomicroscope while still attached to the parent stem, and allowed to slowly dehydrate in air. The target leaf was illuminated homogeneously from below at an intensity of about 40–80 µmol quanta to produce a transmitted light image which was captured on a digital camera (EOS 5D mark III, Canon Cor, Japan) attached to the stereomicroscope. Photographs were taken every 60 s until leaf browning was observed after 26 h. Analysis of the image sequence captured during the drying cycle was carried out to identify rapid changes in light transmission through the leaf veins that corresponded to air entry into the xylem conduits. A similar function ‘Timelapse

Colour Coder' was used in some cases to produce color maps of the timing of embolism throughout the vein network. The count of embolized pixels was then summed to give the cumulative number of pixels present in each vein order over time. The product is a temporally and spatially resolved data set indicating the accumulation of embolisms in the vein orders. Past studies have shown minor veins (higher order than 4°) to be much more resistant to embolism formation than major veins (1°, 2°, 3°, 4° - see description of vein orders below), and the decrease in xylem hydraulic conductance to be mainly driven by drops in conductance of the midrib (Scoffoni *et al.*, 2017). While branches were dehydrating, the Ψ_{leaf} of adjacent leaves was measured using the pressure chamber technique (PMS, Albany, NY, USA) every 1–2 h until the end of the scanning after 26 h of dehydration. In all species, after stomatal closure, the rate of decrease in Ψ_{leaf} was highly linear with dehydration time, therefore it was possible to estimate the Ψ_{leaf} of a target leaf at any time during the drying cycle (Brodribb *et al.*, 2016). By converting time into Ψ_{leaf}, an optical vulnerability curve was produced for the 1° and 2° veins of each leaf, and the Ψ_{leaf} at which leaf conduits reached 12% cumulative embolism (quantifying the initial embolism formation; PLC_{major} P_{12}) and 50% cumulative embolism (an index of resistance to embolism formation; PLC_{major} P_{50}) in 1° + 2° vein orders (quantified by changes in pixel coloration) were obtained for three leaflets from different individuals per species.

Leaf vein density and anatomy

Vein densities were determined as described by Sack *et al.* (2012). Briefly, six leaflets from different individuals of each species were fixed in formalin-acetic acid solution (48 % ethanol: 10 % formalin: 5 % glacial acetic acid: 37 % water), and cleared in 5 % sodium hydroxide in water or ethanol followed by sodium hypochlorite bleach, and stained with safranin and fast green. Stained leaflets were digitally scanned at 9600–12800 dpi (WinRHIZO Root Analysis, Regent Instruments, Quebec, Canada). The leaflets had up to five vein orders: major veins consisted of the midrib (1° vein order), secondary veins branching from the midrib (2°), and tertiary veins connected secondary and primary veins (3°). Minor veins consisted of fourth (4°) and fifth order veins (5°). Whole leaflet images were measured for leaflet area along with the length of the different vein orders using image J (version 1.42q, National Institutes of Health, USA). The major vein density was calculated as the sum of lengths of the 1°, 2° and 3° veins divided by leaflet area. Minor veins were imaged in the top, middle and bottom thirds of the left and right side of each leaflet with a microscope (Ex30LED, Sunnu Instruments, Ningbao, China).

The lengths of the minor veins (4° and higher order veins) were measured in the six segments and averaged (Sack *et al.*, 2012). Minor vein density was calculated according to standard protocol (Scoffoni C, Sack L and PrometheusWiki contributors. "PROTOCOL: Quantifying leaf vein traits." PrometheusWiki).

The anatomical dimensions of the xylem in the leaflets were determined as described by Blackman *et al.* (2010) and Fang *et al.* (2014) using the Ex30LED Sunnu microscope above. Briefly, six compound leaves from different individuals of each species were cut, and immediately scanned to calculate individual leaflet area using Image J software (version 1.42q, National Institutes of Health, USA). Then leaf sections were cut using a freeze-microtome, stained with 5 % toluidine blue, and mounted on glass microscope slides in phenol glycerine jelly to measure the entire area of the xylem in cross sections at the basis of midrib (1°) veins. Midrib xylem area per leaflet area were calculated.

References

Blackman CJ, Brodribb TJ, Jordan GJ. 2010. Leaf hydraulic vulnerability is related to conduit dimensions and drought resistance across a diverse range of woody angiosperms. *New Phytologist* **188**: 1113-1123.

Brodribb TJ, Skelton RP, McAdam SA, Bienaimé D, Lucani CJ, Marmottant P. 2016. Visual quantification of embolism reveals leaf vulnerability to hydraulic failure. *New Phytologist* **209**: 1403-1409.

Fang XW, Turner NC, Palta JA, Yu MX, Gao TP, Li FM. 2014. The distribution of four Caragana species is related to their differential responses to drought stress. *Plant Ecology* **215**: 133-142.

Sack L, Scoffoni C, McKown AD, Frole K, Rawls M, Havran JC, Tran H, Tran T. 2012. Developmentally based scaling of leaf venation architecture explains global ecological patterns. *Nature Communications* **3**: 1-10.

Scoffoni C, Albuquerque C, Brodersen CR, Townes SV, John GP, Cochard H, Buckley TN, McElrone AJ, Sack L. 2017. Leaf vein xylem conduit diameter influences susceptibility to embolism and hydraulic decline. *New Phytologist* **213**: 1076-1092.

Song JS, Wang Z, Sun GZ, Gao HW. 2007. Study on the Tissue Culture of Horqin Peashrub. *Acta Agrestia Sinica* **15:** 66-69.

Video S1 Progression of embolism in *C. korshniskii*. Animated version of Fig 6g.

Video S2 Progression of embolism in *C. sinica*. Animated version of Fig 6h.
